# Unravelling Constant pH Molecular Dynamics in Oligopeptides with Explicit Solvation Model

**DOI:** 10.3390/polym13193311

**Published:** 2021-09-28

**Authors:** Cristian Privat, Sergio Madurga, Francesc Mas, Jaime Rubio-Martinez

**Affiliations:** Department of Material Science and Physical Chemistry & Research Institute of Theoretical and Computational Chemistry (IQTCUB), University of Barcelona, C/Martí i Franquès 1, 08028 Barcelona, Spain; cprivat@ub.edu (C.P.); fmas@ub.edu (F.M.)

**Keywords:** constant pH Molecular Dynamics, AMBER, capped tripeptides, oligopeptides, Ramachandran maps

## Abstract

An accurate description of the protonation state of amino acids is essential to correctly simulate the conformational space and the mechanisms of action of proteins or other biochemical systems. The pH and the electrochemical environments are decisive factors to define the effective pKa of amino acids and, therefore, the protonation state. However, they are poorly considered in Molecular Dynamics (MD) simulations. To deal with this problem, constant pH Molecular Dynamics (cpHMD) methods have been developed in recent decades, demonstrating a great ability to consider the effective pKa of amino acids within complex structures. Nonetheless, there are very few studies that assess the effect of these approaches in the conformational sampling. In a previous work of our research group, we detected strengths and weaknesses of the discrete cpHMD method implemented in AMBER when simulating capped tripeptides in implicit solvent. Now, we progressed this assessment by including explicit solvation in these peptides. To analyze more in depth the scope of the reported limitations, we also carried out simulations of oligopeptides with distinct positions of the titratable amino acids. Our study showed that the explicit solvation model does not improve the previously noted weaknesses and, furthermore, the separation of the titratable amino acids in oligopeptides can minimize them, thus providing guidelines to improve the conformational sampling in the cpHMD simulations.

## 1. Introduction

Nowadays, new methods are being developed to improve simulations of biochemical systems using Molecular Dynamics (MD). The strategies can range from the development of new force fields [1] to the design of new, enhanced sampling methods [2,3,4]. Among them, the introduction of pH in MD simulations is becoming increasingly popular [5,6,7,8,9]. In fact, this property is a crucial factor for proteins whose biological function depends on it, either because they have pH-sensitive domains or the mechanism of action of the active sites needs a specific protonation state of the involved amino acids, among other possible scenarios. Despite its importance for an accurate description of the conformational sampling of proteins, until now the effect of pH was generally dealt by assigning the protonation state of the amino acids according to pKa predictions’ methods, such as H++ [10] or PROPKA [11], on initial conformations, among other approaches [12]. However, this approximation is not very accurate since the prediction is based on a fixed, usually initial, conformation and does not contemplate other conformations because of the time evolution. Treating the protonation state as a dynamic property is very important since the electrochemical environment of the amino acid changes over time and, therefore, changes the *effective* pKa.

To address this issue, many techniques have been developed including the effect of pH in MD simulations in the recent decades, but among them the “constant pH Molecular Dynamics” (cpHMD) approach [13,14,15] has stood out. It has been distinguished according to the strategy to change the protonation station: On one hand, there is the *continuous* cpHMD method [16,17,18,19], which defines the protonation state of proteins with a protonation coordinate λ that is included in the potential function of the system and, on the other hand, the discrete cpHMD method. The latter is based on stochastics to attempt the change of the protonation state after considering the electrochemical environment and the pH of the solvent. One of the first cpHMD methods using discrete protonation states was developed by Baptista et al. [20], and it implemented Monte Carlo and Continuum Electrics (CE) algorithms. This inspired the design of several new methodologies [20,21,22,23]. However, there is still a long walk in the development of these approaches, but first studies highlighted the success and flaws of predicting effective pKa of amino acids inside protein structures [24,25,26,27,28,29]. These revisions made by the scientific community make possible to guide the efforts to improve these techniques, such as the introduction of enhanced sampling techniques, modification of force fields, or solvent molecules, among others, to overcome the pointed drawbacks [18,23,30,31,32,33,34]. 

In this work, we progressed with our study about discrete cpHMD implemented in AMBER suitcase [23,35]. Our research group previously observed some limitations in terms of the conformational space of the titratable residues when performing cpHMD simulations of capped tripeptides in implicit solvent and compared them with conventional MD (cMD) simulations [36]. We finally concluded that the rough approximation made on the fixed partial charges of backbone atoms in titratable residues led to significant deviations in the conformational sampling of the deprotonated forms of the capped tripeptides. Now, we firstly checked the cpHMD method in explicit solvation by studying the capped tripeptides in simulation boxes with TIP3P [37] water molecules. After the results pointed out that limitations on the deprotonated state of cpHMD method persist, we carried out simulations of oligopeptides with aspartic acids in two positions, (1) separate and terminal and (2) adjacent and central, to evaluate if the position of other titratable amino acids can affect the deviations observed in the tripeptides and, thus, open new strategies for the study of large systems with the cpHMD method. 

## 2. Materials and Methods

In view of the objectives of this work, we prepared two sets of peptides to provide results on the performance of the cpHMD method. The first set of simulations includes the capped tripeptide to assess the limitations reported in our previous article [36], but this time including an explicit solvent in the simulations. The second set includes oligopeptides with two aspartic acids placed in distinct positions of the sequence to study the effect of the distance between titratable amino acids when using cpHMD method.

### 2.1. Capped Tripeptides

**Figure 1:**
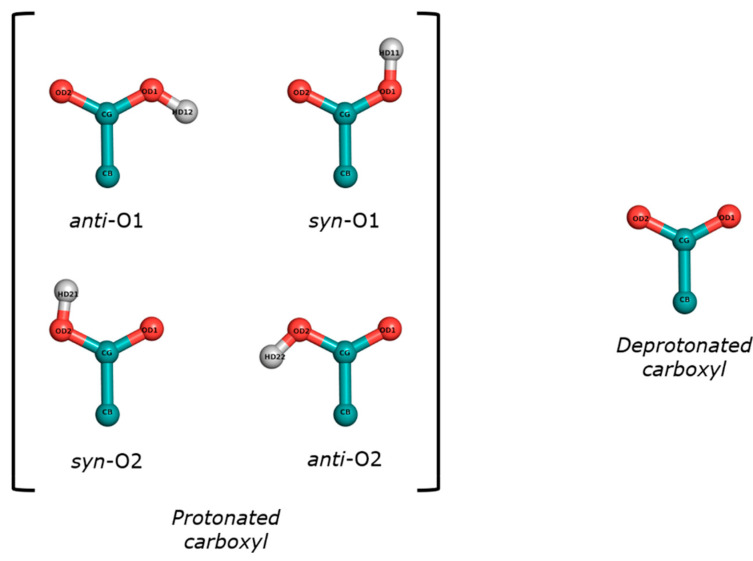
Protonation states of the carboxyl group in the side chains of the AS4 or GL4 residues. There are four protonated states depending on the position (*syn* or *anti*) of the proton with respect to the charged oxygen.

Six tripeptides consisting of two consecutive amino acids with acetyl (ACE) and N-methyl (NME) capping groups on the extremes of the sequence (ACE-X-X-NME) were built using LEaP module of AMBER suitcase. The titratable amino acids available in the cpHMD method of AMBER18 version were Asp (D), Glu (E), His (H), Cys (C), Tyr (Y), and Lys (K). The residues and pH conditions used in the tripeptide simulations are indicated in Table 1. Asp and Glu have special titratable residues (AS4 or GL4) due to the several positions of the proton in the protonated form, as illustrated in Figure 1. The other amino acids use the residue in the protonated form (HIP, CYS, TYR, and LYS) as a titratable residue in the cpHMD method. Since histidine has two protonation states in the neutral form, the delta (δ) and epsilon (ε) protonation states, the corresponding tripeptides in these states were prepared using the HID or HIE residues, respectively, in the cMD simulations. Tyrosine does not parameterize the deprotonated form in the cMD method, so it was not simulated in this protonation state. Finally, a simulation box with a minimum distance of 14.0 Å from the tripeptides to its limit was constructed and filled with TIP3P water molecules. If necessary, counterions were added until the net charge of the simulation box was neutralized. Any solvent molecule closer than 1.0 Å to the solute was rejected to avoid overlapping between molecules. 

### 2.2. Oligopeptides

The second set of simulations was oligopeptides consisting of a lineal chain of eight alanine interrupted by two aspartic acids in distinct positions: (1) adjacent and central (ACE-A-A-A-A-D-D-A-A-A-A-NME or A_4_D_2_A_4_) or (2) separated and terminal (ACE-D-A-A-A-A-A-A-A-A-D-NME or DA_8_D). The ACE and NME capping groups were added in the extremes of the peptides, as we specified in the sequences. The systems were defined as cubic boxes of 77.5 Å per axis and filled with TIP3P water molecules to solvate them. If necessary, counterions were added until the net charge of the simulation box was neutralized. Any solvent molecule closer than 1.0 Å to the solute was rejected to avoid overlapping between solute and solvent molecules.

### 2.3. Preparation of the Input Peptide Structures

The ff14SB force field and constph.lib (the latter only in cpHMD simulations) were loaded in LEaP module to parameterize the capped tripeptides and the oligopeptides. Subsequently, *cpinutil.py* script prepared the protonation states of the titratable residues using GB model of Onufriev et al. [38] (igb = 2) for an ionic strength of 0.1 M. The AS4 and GL4 residues were defined as *syn*-O2 protonated state at acidic pH conditions and the HIP residue started as δ-state at basic pH conditions. The script also modified the intrinsic radii of the carboxylate oxygen in the topology file of those peptides that contained AS4 and GL4 residues [34].

### 2.4. All-Atom Conventional and Constant pH Molecular Dynamics Simulations 

We minimized all peptide systems using the steepest descent method in three stages of restriction. We applied restrictions with a force constant of 5 kcal/mol·Å^2^ in (1) all atoms, (2) backbone atoms, and (3) without restrictions, for 5000 steps at each restriction level. In the cpHMD simulations, we did not turn on the protonation state change attempt during the minimization. Next, we heated up the systems from 0 up to 300 K with a lineal increase of 1 K·ps^−1^ in the NVT ensemble and subsequently equilibrated during 200 ps in the NPT ensemble. Using the last coordinates after preparing the peptide systems, we generated four replicates with random initial velocities, following a Maxwell–Boltzmann distribution, and carried out production runs of 500 ns (4 replicates × 500 ns = 2 µs per simulation conditions) in the NVT ensemble in order to increase the conformational sampling [39]. A Langevin thermostat was set up with a collision frequency of 3 ps^−1^. Periodic boundary conditions and SHAKE algorithm were applied in the simulations. In the cpHMD simulations, explicit cpHMD method was turned on with a protonation state change frequency of 0.2 ps⁻^1^ and water molecules were relaxed 0.2 ps after a successful trial. We ensured fully protonated or deprotonated states of the titratable amino acids by setting strong acidic (pH = 1) and basic (pH = 12) pH conditions in the cpHMD simulations. Titratable LYS residues needed more basic conditions (pH = 14.0). In the oligopeptide simulations, we fixed pH conditions at 10.0 for a fully deprotonated state since Asp amino acids have a low intrinsic pKa. The simulations carried out in this study are collected in Table 1. All the MD calculations were done with GPU version of PMEMD software.

### 2.5. Energetic and Conformational Analysis

In all simulations, we saved the conformational configurations in the trajectory file every 10 ps. Energy contributions were later recalculated with a cutoff of 10.0 Å and using trajectories without solvent water molecules. Electrostatics were determined via Particle Mesh Ewald with a long-range correction for periodicity. Additionally, we also computed the electrostatics dividing the capped tripeptides into backbone atoms, including capping groups, and side chain atoms. In cpHMD simulations, we also obtained the protonation fractions and the populations of each protonation state of the titratable amino acids using *cphstats* implemented in AMBER to check that cpHMD simulations were carried out in fully protonated or deprotonated states.

The radial distributions functions (RDFs) and φ and ψ dihedral angles of the tripeptides were calculated with the CPPTRAJ module. Radial distribution functions were computed using the distance of the water molecules around specific atoms of the side chains of each amino acid. An in-house script built the Ramachandran maps by transforming the dihedral angles’ data into Gibbs free energy, as indicated in Equation (1).

(1) $$∆G=-k_{b}Tln\left(n_{i}/n_{max}\right)$$

where *k_b_* is the Boltzmann constant, *T* is the temperature, and *n_max_* and *n_i_* are the maximum population and the population of a cell *i* in a grid of the dihedral angles with a spacing of 1°. We classified the regions of the Ramachandran maps according to Rubio et al. [39], as illustrated in Figure S1.

The conformational properties of oligopeptides were analysed by means of radius of gyration (Rg) and secondary structure fractions (fSS) using CPPTRAJ module. We calculated the R_g_ using C_α_ atoms and the fSS with the DSSP method applied in all backbone atoms. All trajectories were superimposed to the lineal conformation to perform the Principal Component Analysis (PCA) using the covariance matrix as a metric. Conformational configurations were projected in the Principal Component (PC) space to calculate the Gibbs free energy, as indicated in Equation (1) with a spacing of 0.2°. Finally, we clustered the trajectories with the hierarchical agglomerative (bottom-up) approach using the root mean square displacement (RMSD) of the C_α_ atoms as a distance. Conformational configurations were divided into 15 clusters with a sieve of 20 frames. Then, we calculated the RMSD between all the representative conformations of each cluster (2D RMSD). All plots were generated with GNUPLOT (version 5.2).

## 3. Results

### 3.1. Capped Tripeptides with Explicit Water Molecules

Firstly, we simulated the capped tripeptides in explicit water molecules using cMD and cpHMD methods. Ramachandran maps of the capped tripeptides were built by representing the φ and ψ backbone dihedral angles of each of the two monomers (the N- or C-terminal amino acid) of the tripeptide. We divided these maps into nine regions, defined in Figure S1 according to the predominant conformation previously described by our research group [36]. The populations of each conformational region were calculated to provide a conformational profile of each simulated peptide. In addition, the distributions of each energy contribution were plotted, and the electrostatics were recalculated stripping water molecules. In this study, we focused on the latter contributions, which are fundamental in the change of the protonation states of the titratable amino acids. Finally, the effect of the electrostatics’ interactions on the solvent molecules was studied by radial distribution functions (RDFs) of water molecules around the tripeptides. 

Capped Asp tripeptide is mainly discussed in this section to assess the strengths and weaknesses of the acidic amino acids in the cpHMD method when the explicit solvation model was introduced in the simulations. We previously carried out a study of these capped tripeptides but using the implicit solvent model, reporting inconsistencies on the approach used to assign partial charges, among other possible artifacts [36]. In this work we wanted to elucidate if these limitations of the cpHMD method persist when the solvent is simulated explicitly. We will also discuss the evidence observed in the other tripeptides, the results of which are reported in the SI.

#### 3.1.1. Conformational Sampling Disagrees in Deprotonated Forms of Amino Acids with Several Protonation States

The combinations of ψ and φ dihedral angles of each amino acids were represented in the Ramachandran maps to obtain a profile of the secondary structure of each amino acid in the capped tripeptides. Thus, we delineated a grid on these maps to get the population fraction of each bin and, thus, calculate the Gibbs free energies. In addition, we measured the populational ratios of the nine conformational regions, as we detailed in Methods.

**Figure 2:**
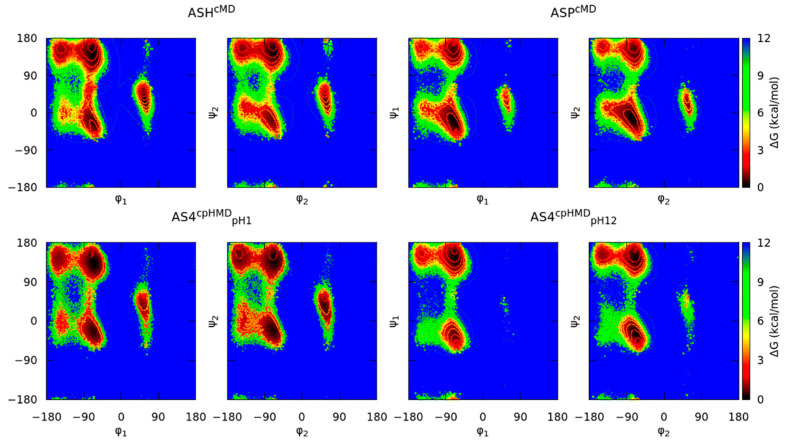
Ramachandran maps of the capped Asp_2_ tripeptide. Titles indicate the residues, the simulation method in the superscripts, and the pH value in the subscripts. Each simulation condition has two energy maps according to the set of backbone dihedral angles of the N-terminal (φ_1_/ψ_1_) or the C-terminal amino acid (φ_2_/ψ_2_). Solid lines indicate an increase of 0.6 kcal/mol in the energy map.

**Figure 3:**
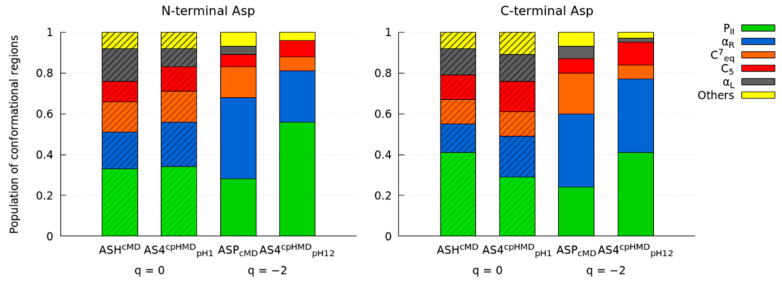
Populations of the conformational regions (P_II_, α_R_, C^7^_eq_, C_5,_ and α_L_) in the Ramachandran maps of each amino acid of the capped Asp_2_ tripeptide. Labels indicate the residues, the simulation method in the superscripts, and the pH value in the subscripts. Net charge of the tripeptide was below (q). Striped or solid box style are protonated or deprotonated states, respectively.

The Ramachandran maps of the capped Asp_2_ tripeptide are illustrated in Figure 2. In the protonated form of Asp tripeptide, the conformational profiles of the cMD (ASH^cMD^) and cpHMD (AS4^cpHMD^_pH1_) simulations did not completely satisfy the minima of the main populated regions (P_II_, α_R_, C_7_^eq^, and C_5_) and, furthermore, neither did the shapes of the α_R_ region. We also observed this behaviour in the energy maps of the deprotonated form (ASP^CMD^ and AS4^CPHMD^_pH12_), in which, again, the minima and the shape of the α_R_ region did not correspond between the methods. We illustrated the population of the main conformational regions to quantitatively compare between the simulation methods in Figure 3. Here, the protonated form (ASH^CMD^ and AS4^CPHMD^_pH 1_) showed mild differences (about ~10% maximum) in the populations of the regions, which can be accepted within a tolerance due to differences in the protonation state sampling between the methods. Thus, despite the deviation on the minima, the population profiles of the protonated simulations were generally in agreement. However, when the Asp_2_ tripeptide was deprotonated (ASP^CMD^ and AS4^CPHMD^_pH12_), the systems presented stronger dissimilarities on the conformational regions. A low population ratio of α_L_ conformation confirmed the region was not sampled in the deprotonated cpHMD system.

In the current study, we also carried out the simulations of each titratable amino acid available in AMBER. On one hand, there were the hydrophilic Glu (acidic) and His (basic) amino acids. The former is structurally like the Asp amino acid but with an additional methyl group in the side chain (and a slight shift in the intrinsic pKa). Indeed, the Ramachandran maps of the protonated (GLH^cMD^ and GL4^cpHMD^_pH1_) and deprotonated forms (GLU^cMD^ and GL4^cpHMD^_pH12_) showed a similar behaviour as the Asp_2_ tripeptide in Figure S2. The conformational populations corroborated it (Figure S3), in which the populations of the conformational regions in the Ramachandran maps exhibited a clear disagreement in the deprotonated form. The case of histidine is more interesting since this amino acid has a side chain with an imidazole ring that can be defined as N-delta nitrogen (δ) or N-epsilon nitrogen (ε) protonated in the neutral form. Differently to the previous amino acids, histidine became doubly protonated (and positively charged) in the imidazole ring when it was found in the protonated form. The Ramachandran maps of the capped His_2_ tripeptide showed a good agreement to the protonated form (HIP^cMD^ and HIP^cpHMD^_pH 1_) (Figure S4). The population ratios of the regions showed this trend (Figure S5). However, the neutral δ- and ε- states of the His_2_ tripeptide were remarkably different in cpHMD (HIP^cpHMD^_pH 12_) from cMD (HID^cMD^ and HIE^cMD^). HIP^cpHMD^_pH12_ showed a singular conformational profile in the Ramachandran maps and populations ratios, which were, surprisingly, more similar to the protonated form rather than the neutral HID^cMD^ or HIE^cMD^ tripeptides.

On the other hand, the hydrophilic basic Lys, the hydrophobic aromatic Tyr, and the hydrophilic polar Cys constituted a set of amino acids with titratable groups in the side chains with intrinsic pKa values > 7.0 (i.e., 10.4, 9.6, and 8.5, respectively). The protonated form of lysine (LYS^cMD^ and LYS^cpHMD^_pH1_), tyrosine (TYR^cMD^ and TYR^cpHMD^_pH1_), or cysteine (CYS^cMD^ and CYS^cpHMD^_pH1_) in the capped tripeptides showed closer conformational profiles in the Ramachandran maps (Figures S6–S8) as well as the populations of the conformational regions (Figures S9–S11) when the simulation methods were compared. The conformational sampling of the cysteine was also in agreement in the deprotonated form (CYM^CMD^ and CYS^CPHMD^_pH12_) of both methods. However, the deprotonated form of lysine (LYN^CMD^ and LYS^CPHMD^_pH14_) showed mild but not significant differences in the conformational profile in the Ramachandran map and the population ratios. We remember that tyrosine in the deprotonated form was not evaluated in this work due to it not being parameterized in the classic ff14SB, although the partial charges of the side chain atoms in the deprotonated state were available in cpHMD libraries.

The conformational profiles of the capped tripeptides showed that conformational samplings of the deprotonated forms generally disagreed when simulation methods were compared, except for those amino acids with pKa > 7, in which the differences were very low or acceptable with a tolerance. The protonated forms of the amino acids in tripeptides agreed in the conformational samplings even though those with several protonation states (Asp, Glu, and His) exhibited negligible shifts in the populations of the conformational regions. The inclusion of TIP3P water molecules generally led to an increase of the P_II_ population, except for a few specific cases, but persisted in the differences between the simulation methods that were reported in our previous study with implicit solvation model. In that report, we mainly attributed the noted discrepancies in the conformational sampling to a rough approach in the sets of partial charges in the backbone and beta carbon (C_β_) atoms, among other minor reasons. Thus, despite the inclusion of explicit water molecules, the inconsistencies noted in the deprotonated form did not improve when comparing the simulation methods.

#### 3.1.2. Energy Contributions Reveal Deficiencies in the Reproduction of Electrostatics’ Interactions

Each energy term involved in the simulations was calculated using the CPPTRAJ module and then we performed the normalized distributions to compare the performance of each simulation method. With respect to electrostatic interactions, we calculated the 1–4 and long-range interactions in two situations: (1) with TIP3P water molecules and (2) ignoring the solvent. Despite this work only illustrating electrostatics without solvent molecules, both cases were considered to discuss the following section. This decision was made since the large proportion of solvent–solvent interactions resulted in a masking effect to observe the consequences of the approach in the backbone partial charges of the titratable amino acids. To assess the effect of the electrostatics on the solvent, we calculated the RDF of the water molecules around each amino acid.

As can be seen in Figure S12, the energy distributions of the Asp tripeptide showed dissimilarities in both protonated (ASH^cMD^ and AS4^cpHMD^_pH1_) and deprotonated forms (ASP^CMD^ and AS4^CPHMD^_pH12_) in electrostatics, dihedral, and angular contributions. The protonated form had electrostatics’ distributions in a close energy range but with distinct shapes while the deprotonated form presented distributions in far energy ranges. The cpHMD simulations shared angular and dihedral distributions, independently of the pH conditions, and were not coincident with the cMD ones. Additionally, we computed the electrostatic distributions including only the backbone or the side chain atoms to understand the effect of the inaccurate assignment of the partial charges in the simulation (Figure S13). In the protonated form (ASH^cMD^ and AS4^cpHMD^_pH1_), the backbone electrostatics agree in both 1–4 and long-range terms. However, the electrostatics of the side chain atoms failed in the shape of the distributions. The deprotonated form (ASP^cMD^ and AS4^cpHMD^_pH12_) exhibited mild shifts but similar shapes in both backbone and side chain electrostatics’ distributions. Even so, we noted that backbone electrostatics of the deprotonated form (AS4^cpHMD^_pH12_) were closer to the protonated ones (ASH^cMD^ and AS4^cpHMD^_pH1_) rather than the ASP^cMD^. 

The energy contributions behaved similarly to the simulations with implicit solvation model. Except for some differences in the electrostatics, which may be expected since the explicit solvation model is more accurate, the reported inconsistencies were also evidenced in our previous work. We explained there that the failure on reproducing the electrostatics in the deprotonated form was mainly due to the approach on the partial charges in the cpHMD, in which we assigned and fixed the partial charges of backbone of the ASH residue to the AS4 residue, independently of the protonation state. The partial charges of the side chains’ atoms in cpHMD method were the corresponding ones to those in the protonation state in the cMD method, but adjusting the value of C_β_ atom to ensure a change in the net charge of ±1 when the other protonation state was accepted. For this reason, the electrostatics of the deprotonated form did not match in the backbone, in which the cpHMD simulations had distributions like the protonated form. Other factors were involved in these inconsistencies, e.g., the definition of dummy hydrogen atoms as ghost atoms or the distinct sampling of the protonation state during the simulations. Note that the protonated form in the cpHMD method started in the syn-O2 protonation state but rapidly changed into other protonated states over time after accepting a criterion. At the end of the simulations, we calculated the populations on each protonation state and found that AS4^cpHMD^_pH1_ was mainly populated in the syn-O1 (47.2 and 45.7%) and syn-O2 protonated states (47.1% and 45.9% for each amino acid, respectively). The change of protonation state in the cMD simulations was slower because it was achieved through the rotation of the bonds and angles of the carboxyl groups. In our previous work, we suggested that this faster sampling of the protonation states was probably the reason for the deviations observed in the conformational profiles. It should be studied if this represents an improvement in the conformational sampling of these peptides.

**Figure 4:**
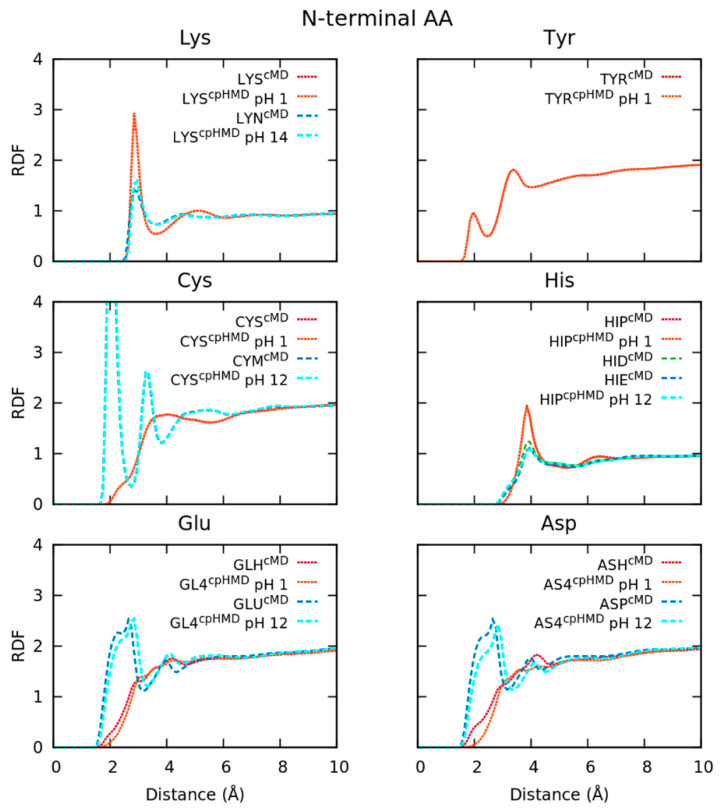
Radial distribution functions (RDFs) of the solvent water molecules around each amino acid of the capped tripeptides. Only the N-terminal amino acid of each tripeptide structure is shown in the plot. Simulations in the protonated form are represented with dotted lines while the deprotonated ones are in dashed lines.

The radial distribution function (RDF) of TIP3P water molecules around the capped tripeptides was calculated to understand the effect of the partial charges in the solute–solvent electrostatics’ interactions. The RDF profiles of the Asp_2_ tripeptide were in good agreement in both protonated and deprotonated forms in Figure 4. The former (ASH^cMD^ and AS4^cpHMD^_pH1_) showed smooth changes in the shape of the distribution, while the deprotonated form (ASP^cMD^ and AS4^cpHMD^_pH12_) only showed a mild shift. However, these deviations seemed to be not significant. The N-terminal or C-terminal position of the Asp amino acid in the tripeptide sequence did not apparently influence the RDFs.

Back to other titratable amino acids, the Glu tripeptide showed a similar behaviour as the Asp tripeptide in the energy distributions (Figures S14 and S15). Electrostatic interactions as well as dihedral and angular energies did not correspond between methods for both protonated (GLH^cMD^ and GL4^cpHMD^_pH1_) and deprotonated (GLU^cMD^ and GL4^cpHMD^_pH12_) forms. The case of the His tripeptide was more challenging since the δ- and ε-neutral forms were fixed in the cMD simulations, while the cpHMD method allowed the exchange between both states. Thus, HID^cMD^ and HIE^cMD^ cannot be strictly compared to HIP^cpHMD^_pH12_. All energy contributions of the protonated form (HIP^cMD^ and HIP^cpHMD^_pH1_) agreed, as can be seen in Figure S16. Instead, the deprotonated form (HID^cMD^, HIE^cMD,^ and HIP^cpHMD^_pH12_) did not correspond when comparing simulation methods but showed total energy distributions in a close range. Only electrostatics’ interactions had remarkable shifts between cpHMD and cMD simulations. Note that we computed the energy distributions of HIP^cpHMD^_pH12_ fixing the set of partial charges in the side chains’ atoms from one of two protonation states, which were very rough approximations. Thus, the energy distributions of HID^cMD^ and HIE^cMD^ were delimiters of the energy range in which the distributions of HIP^cpHMD^_pH12_ should be located. We also divided the electrostatics into backbone and side chain atoms (Figure S17). The backbone electrostatics of HIP^cpHMD^_pH12_ were overlapped to the protonated form, as observed in other peptides. The sidechain electrostatics presented mild shifts with respect to the HID^cMD^ and HIE^cMD^ simulations, suggesting that the source of the deviation was mainly due to the failure to reproduce the backbone electrostatics. 

Despite exhibiting deficiencies in the electrostatics, the RDFs of each protonation form of the capped Glu and His tripeptides showed good overlapping in Figure 4. Glu tripeptides had mild shifts in the protonated (GLH^cMD^ and GL4^cpHMD^_pH1_) and deprotonated (GLU^cMD^ and GL4^cpHMD^_pH12_) forms. In the His tripeptides, the RDFs of the simulations in the protonated form (HIP^cMD^ and HIP^cpHMD^_pH1_) perfectly overlapped, and the deprotonated HIP^CPHMD^_pH12_ also did with HIE^cMD^ and HID^cMD^. 

Finally, the energy contributions of the protonated form of Lys (LYS^cMD^ and LYS^cpHMD^_pH1_), Tyr (TYR^cMD^ and TYR^cpHMD^_pH1_), and Cys (CYS^cMD^ and CYS^cpHMD^_pH1_) tripeptides agree, as can be seen in Figures S18–S20. However, the deprotonated form of Lys (LYN^cMD^ and LYS^cpHMD^_pH14_) presented shifts in the distributions of the electrostatics and, therefore, in the total energies. The deprotonated Cys tripeptide (CYM^cMD^ and CYS^cpHMD^_pH12_) also failed to overlap the electrostatics’ energies and, in addition, the dihedral energies. When we divided the electrostatic interactions into backbone and side chains’ atoms, the protonated forms of the tripeptides with pKa > 7.0 perfectly overlapped in all electrostatics contributions (Figures S21–S23). Nonetheless, the deprotonated forms showed mild shifts in the side chain electrostatics and the backbone electrostatics of the cpHMD simulation overlap to the protonated distributions. The RDFs in Figure 4 show perfect overlapping in all forms, suggesting the approach in the set of partial charges of the backbone atoms did not influence the distribution of water solvent molecules around the tripeptides.

Because of the explicit description of the solvent molecules in the simulations, smooth changes in the shape or the energy range of the distributions were appreciated when we compared with the simulations with the implicit solvent. Even so, all the simulations, independently of the solvation model, showed similar trends. It should be noted that His amino acid could not be compared because we used a simple approximation to calculate the electrostatic energy and, therefore, the distributions in the cpHMD simulation at pH 12 should be averaged over the population of each state (δ or ε). In this case, the ε-state became more populated in the explicit solvation model (30% and 22% for N- and C-terminal amino acids, respectively) in front of the simulations with the implicit solvent (23% and 19%). However, the δ-state still predominated in the strong basic conditions (70% and 78%), which was in line with the evidence observed in the Ramachandran maps and conformational populations.

### 3.2. Titratable Aspartic Acids in Adjacent and Terminal Positions in Oligopeptide

After revising the strengths and weaknesses of the cpHMD method in the tripeptides with explicit solvation, we built an oligopeptides of 8-Ala amino acids and two Asp in (1) separated and terminal (DA_8_D peptide) and (2) adjacent and central (A_4_D_2_A_4_ peptide) positions as models. Through these simulations, we wanted to assess if the failure in the electrostatics or other reported discrepancies of the cpHMD method persisted in these oligopeptides and how the distance between the titratable amino acids can affect the results.

We carried out 8-µs-length simulations for each of these oligopeptides, A_4_D_2_A_4_ and DA_8_D, in the two protonation forms of Asp amino acid in the cMD and cpHMD simulation methods. Next, we examined the conformational sampling of these peptides by clustering the trajectories and building energy maps in the Principal Component (PC) space. Other properties related to the conformational sampling were also computed, such as radius of gyration (R_g_) and secondary structure propensities (fSS). Finally, the distributions of the energy contributions were illustrated to understand the implications of using the cpHMD method on these systems.

#### 3.2.1. Position of the Titratable Amino Acids Modulates the Conformational Sampling

Initially, we used the conformations of the trajectories to create a covariance matrix, build the Principal Components Analysis (PCA) space, and project the conformations in this new space. Then, we computed the Gibbs free energies by generating a grid in the PCA space and calculating the populations of each bin. Approximately 50% of the information of the conformational sampling was collected in these energy maps. More details are specified in Methods.

**Figure 5:**
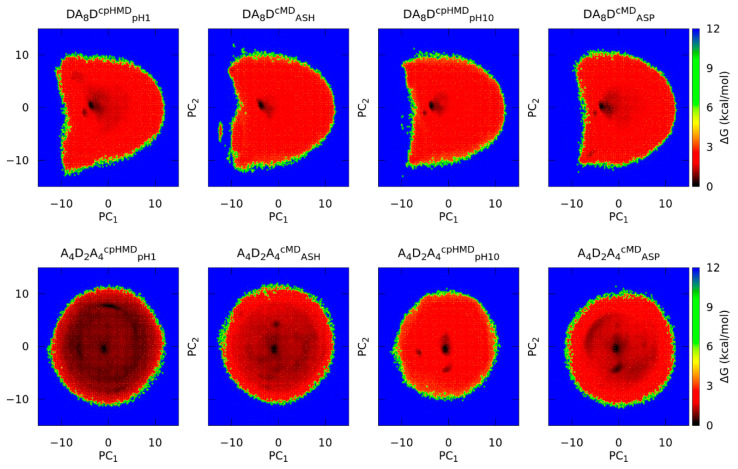
Gibbs free energies in the Principal Component space of the oligopeptides. The four plots in the top correspond to DA_8_D peptide and the four in the bottom correspond to the A_4_D_2_A_4_ peptide. The subtitle of each graph indicates the peptide system, the simulation method (in the superscript), and the residue label or the pH (in the subscript) of the energy map. The PC spaces of the systems were built independently of each other.

**Figure 6:**
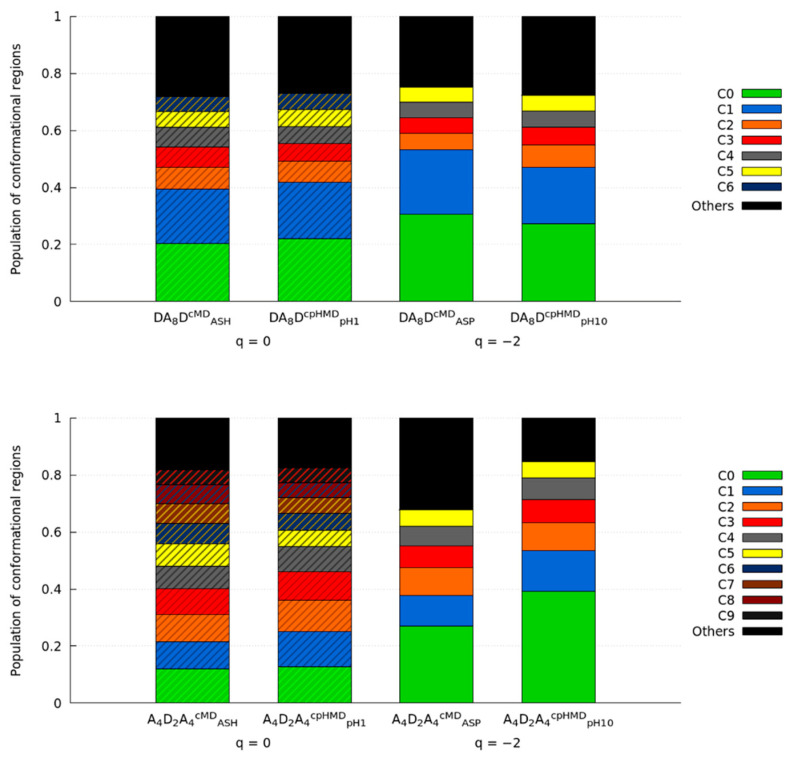
Representation of the clusters with a population ratio > 5% (DA_8_D and A_4_D_2_A_4_ in the top and the bottom, respectively) in all simulation methods. Each system indicates the residue, the simulation method (in the superscript), and the pH (in the subscript, only the CPHMD simulations). Total charge of the tripeptide is indicated below the systems (q = −2, 0). The box style (striped or solid) indicates those systems in the same protonation state, independently of the simulation method.

The energy maps in the PC space of the DA_8_D and A_4_D_2_A_4_ peptides are displayed in Figure 5. The oligopeptide with terminal titratable amino acids (DA_8_D) showed similar conformational sampling independently of the protonation state or even the simulation method. The location of minima or populated regions became more difficult to reproduce in the energy maps. Some subtleties were appreciated in the maps, e.g., the DA_8_D^CPHMD^_pH1_ system was more distributed in the space since a darker area was evidenced or a new minimum appeared in DA_8_D^CMD^_ASH_. To compare the conformational sampling of each system quantitatively, the trajectories were clustered, and the populations of the main clusters are illustrated in Figure 6. These clusters were ordered by the ratio of population, which does not necessarily mean that they corresponded to the same or near regions in the conformational sampling. Both protonated and deprotonated forms showed good agreement on the populations of each cluster when simulation methods were compared. In fact, the populations between protonated and deprotonated oligopeptides were not very different. The 2D-RMSD of the representative conformation of the main clusters was also calculated (Figures S24 and S25) to measure the structural similarity. In the protonated form, the superimposition of the representative conformations of clusters C0 and C1 had a great RMSD value, indicating that DA_8_D^CMD^_ASH_ and DA_8_D^CPHMD^_pH1_ reached similar conformations for, at least, ~40% of the sampling. Other RMSD values showed good fitting between lowly populated clusters. However, the 2D-RMSD of the deprotonated form indicated lower but still good fitting values. The two most populated clusters, C0 and C1, were present in both simulation methods but with changed orders of population. Other exchanges between lowly populated clusters were observed in the 2D-RMSD of the protonated and deprotonated forms, or even some representative conformations that apparently did not fit any other cluster. 

To analyse the convergence of the simulations, we computed the distribution of the first three PCs at 2, 4, and 8 µs of the protonated and deprotonated forms of DA_8_D (Figure S26). In general, the distributions did not change significantly during the reported times, but the observed peaks did. This suggests that more simulation time might be needed until the PC distributions stabilize. Because the peaks at 4 and 8 µs showed small changes, but still important on some systems, we expanded the simulations of DA_8_D up to 10 µs to ensure the convergence. Not-remarkable changes were appreciated when we expanded the simulation from 8 to 10 µs. Thus, we finally concluded that simulation lengths of 8 µs were enough to widely sample the conformational space of the oligopeptides.

On the other hand, the A_4_D_2_A_4_ peptide shows remarkable differences in the energy maps of Figure 5. The protonated form (A_4_D_2_A_4_^cMD^_ASH_ and A_4_D_2_A_4_^cpHMD^_pH1_) is very distributed throughout the conformational space. The conformational populations of the clusters corroborated it since the ratios of the most populated clusters were very high. The 2D-RMSD values were not encouraging since the representative conformation of the most populated cluster (C0) of A_4_D_2_A_4_^cpHMD^_pH1_ did not fit with any of the most populated clusters of A_4_D_2_A_4_^cMD^_ASH_ or the cluster C1 of A_4_D_2_A_4_^cpHMD^_pH1_ corresponding to C4 of the cMD method. Since all clusters had closer populations and good RMSD values were observed in the 2D-RMSD plot and in other clusters, we suggest that conformational sampling could be similar between methods. The simulations of the oligopeptide in the deprotonated form (A_4_D_2_A_4_^cMD^_ASP_ and A_4_D_2_A_4_^cpHMD^_pH10_) showed a more localized conformational sampling (Figure 5), especially for the A_4_D_2_A_4_^cpHMD^_pH10_, which clearly exhibited three minima. Indeed, it showed ~60% more population encompassed the three most populated, if it was compared with the peptide in cMD method, A_4_D_2_A_4_^cMD^_ASP_. However, the RMSD values indicated a good fit between the representative conformations of the clusters of both cMD and cpHMD methods (Figures S27 and S28), suggesting that there were small structural changes.

Thus, we found similar population ratios between clusters and tolerable agreement in the RMSD of representative conformations (but in exchange order) in the protonated form, while the deprotonated form showed better RMSD values between simulation methods but more shifts in the population ratios. To elucidate the behaviour observed in the energy maps and the clusters, we calculated some conformational properties to check if these differences were also observed.

#### 3.2.2. Terminal Titratable Residues Describe Correctly the Conformational Properties

**Figure 7:**
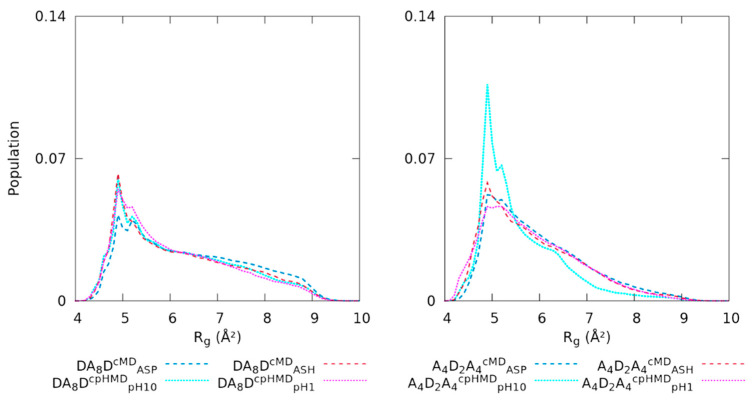
Comparison of the normalized distributions of Rg of the DA_8_D (**left**) and A_4_D_2_A_4_ (**right**) peptides. The simulation methods are represented in dashed (cMD) or dotted (cpHMD) lines. Cyan and blue colours indicate the deprotonated form, and magenta and red colours indicate the protonated one.

To elucidate other implications on the conformational sampling that could depend on the simulation method, we calculated the radius of gyration of the peptides to measure their compactness and illustrated them in Figure 7. The R_g_ distributions of DA_8_D peptide were in good agreement in both protonated and deprotonated forms (Figure 7), except for the two peaks located at ~5 Å. Even so, the protonated form (DA_8_D^cMD^_ASH_ and DA_8_D^cpHMD^_pH1_) was reasonably similar on the first peak while the deprotonated form (DA_8_D^cMD^_ASP_ and DA_8_D^cpHMD^_pH10_) only overlapped on the second one. Thus, the DA_8_D peptide was consistent between simulation methods, but smooth deviations in the peaks were observed. On the other hand, the A_4_D_2_A_4_ remarkably disagreed on the deprotonated form (A_4_D_2_A_4_^cMD^_ASP_ and A_4_D_2_A_4_^cpHMD^_pH10_) of the cpHMD simulation. The tail of the R_g_ distribution of A_4_D_2_A_4_^cpHMD^_pH10_ decayed and the first peak was larger than the cMD simulation, suggesting more compacted conformations with respect to A_4_D_2_A_4_^cMD^_ASP_. The protonated form (A_4_D_2_A_4_^cMD^_ASH_ and A_4_D_2_A_4_^cpHMD^_pH1_) had similar distributions but with a mild shift in the first peak. The deviation in the deprotonated form was in line with the analysis of the conformational sampling observed in the energy maps and the clusters.

**Figure 8:**
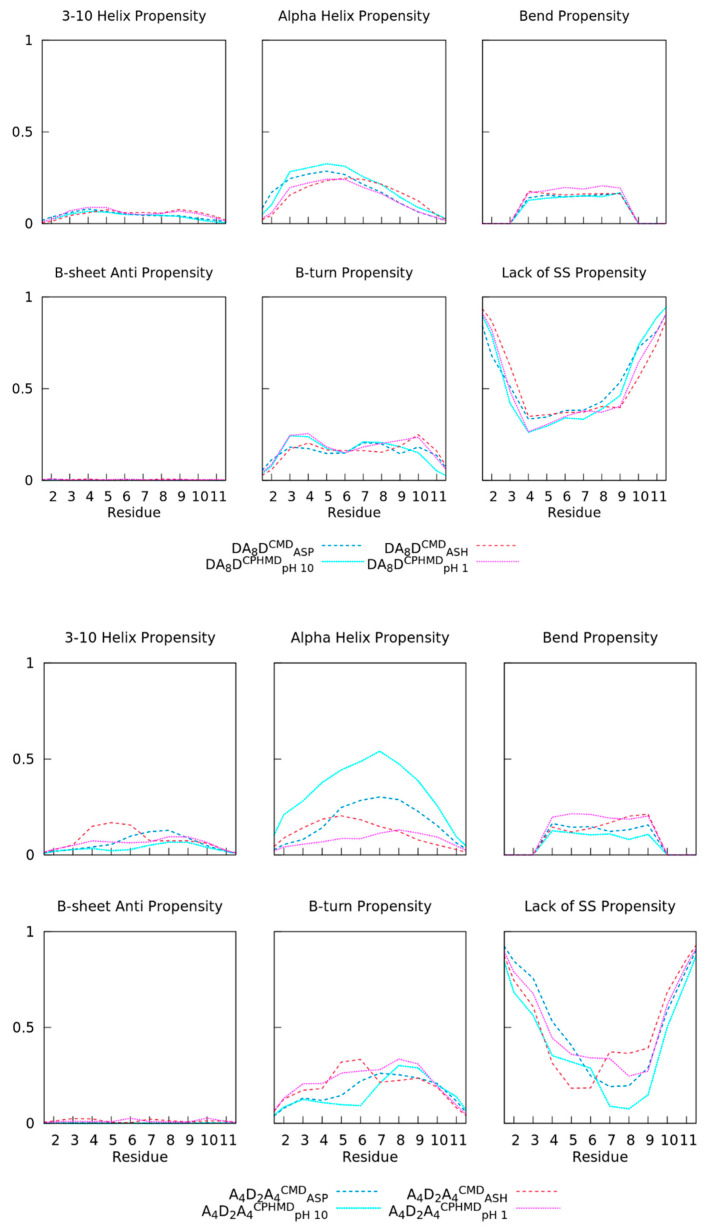
Propensity factors of the secondary structure (fSS) of each amino acid in the DA_8_D (**top**) and A_4_D_2_A_4_ (**bottom**) oligopeptides using the DSSP algorithm. Para Β-sheet and π-helices were omitted due to their absence. Dashed and dotted lines correspond to fixed (cMD) or dynamic (cpHMD) protonation sampling during the simulation.

Furthermore, the secondary structure propensities (fSS) of the peptides were calculated by using the DSSP method. In Figure 8, the DA_8_D peptide shows good agreement between both forms within a tolerance of ~5%. The bend and 3_10_ helix SS greatly overlapped when simulation methods were compared, while other propensities (α-helix, bend, or β-turn) showed mild but not significant deviations in the fractions. The propensity of forming a random coil structure was larger (~35%) in front of structuring helices (~25%) or other secondary structures. On the other hand, the A_4_D_2_A_4_ peptide was more diverse in terms of the fSS propensities. Neither the protonated nor the deprotonated forms overlapped in the cMD and cpHMD simulations, even for those SS (beta and 3_10_ helix) with low propensity. Generally, deviations of up to 20% were observed in the propensities, except for the deprotonated form (A_4_D_2_A_4_^cMD^_ASP_ and A_4_D_2_A_4_^cpHMD^_pH10_) that stood out in the alpha helix conformation. The high propensity of forming α-helix in the A_4_D_2_A_4_^cpHMD^_pH10_ was in line with the high compactness found in the R_g_ distribution. Thus, the conformational properties of the peptides with adjacent titratable Asp highlighted greater deviations on the fSS propensities and R_g_ that apparently depend on the simulation method.

#### 3.2.3. Different Description on Electrostatics and Dihedral Energies Causes Deviations in Conformational Sampling and Properties

To comprehend the origin of the deviations on the conformational sampling and properties, now we focus on the energy contributions of the simulations. We recalculated the intra- and intermolecular energies, but previously stripping the solvent molecules and calculating their normalized distributions. We also considered the energy distributions of the simulations with the solvent molecules.

**Figure 9:**
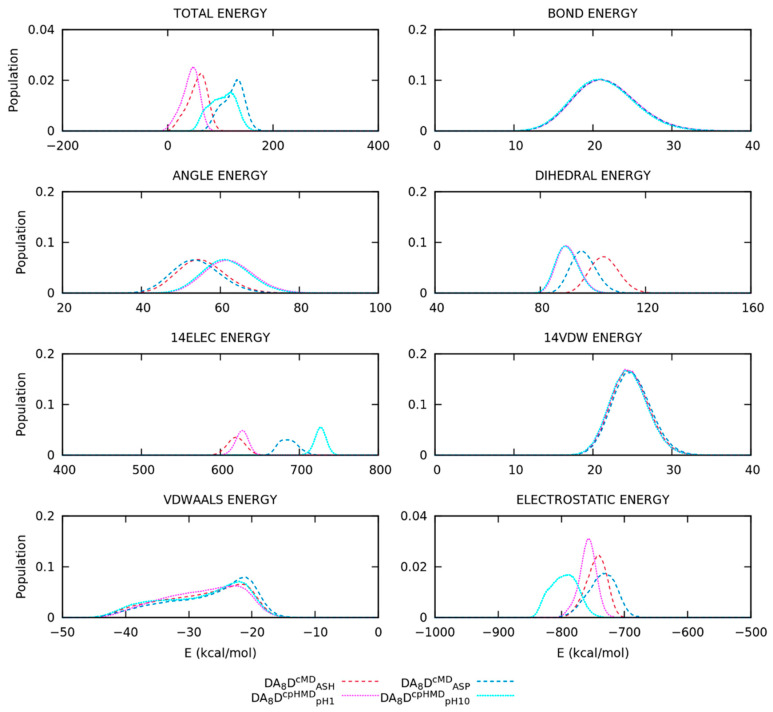

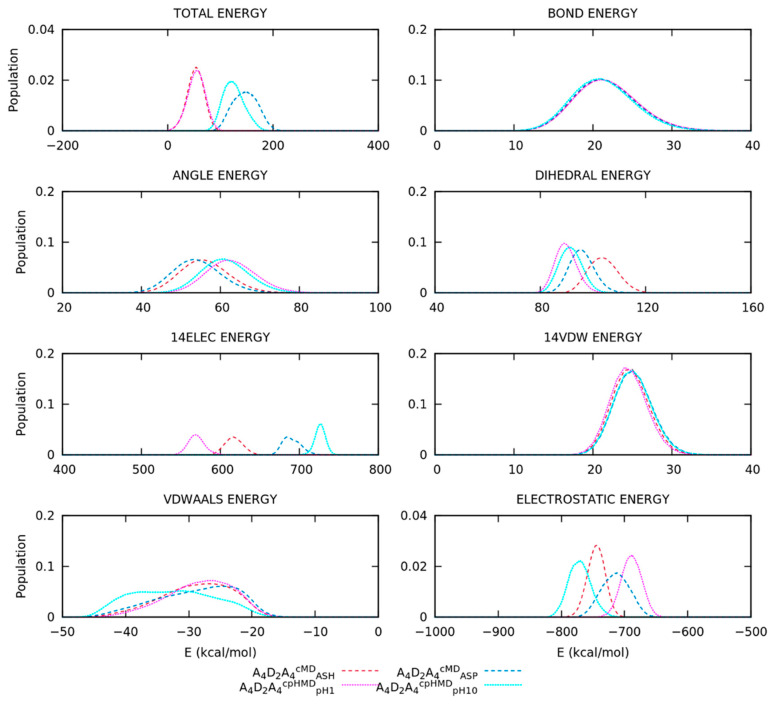
Normalized distributions of total, intra-, and intermolecular energies of the oligopeptides. The first set of plots in the (**top**) corresponds to the DA_8_D peptide and the second set in the (**bottom**) corresponds to the A_4_D_2_A_4_ peptide. The simulation methods are represented in dashed (cMD) or dotted (cpHMD) lines. Water molecules were not included in the calculations.

The total energy of the DA_8_D peptide showed mild shifts in the distributions of the cMD and cpHMD simulations due to the disagreement of the angular, dihedral, and electrostatics’ (1–4 and long-range) contributions (Figure 9). On one hand, the electrostatics of the protonated (DA_8_D^cMD^_ASH_ and DA_8_D^cpHMD^_pH1_) and deprotonated (DA_8_D^cMD^_ASP_ and DA_8_D^cpHMD^_pH10_) were in a close energy range but exhibited different shapes in the distributions. The cpHMD simulations had a large peak on electrostatics while the cMD ones showed wider distributions. On the other hand, the distributions of the angular and dihedral energies overlapped in the cpHMD simulations, independently of the pH. This evidence was observed and repeated in previous systems, i.e., tripeptides in explicit and implicit solvation model. Finally, the simulation methods showed smooth deviations in the energy distributions of the DA_8_D peptide, especially in the deprotonated form, which agreed with the behaviour observed on the conformational properties (R_g_ and fSS). However, the inconsistencies of the cpHMD residues were not enough to significantly modify the conformational space of the DA_8_D peptide or, in other words, when the cpHMD residues were separated in the peptide sequence.

The A_4_D_2_A_4_ peptide, on the other hand, showed a good overlapping on the distributions of the total energy of the protonated form (A_4_D_2_A_4_^cMD^_ASH_ and A_4_D_2_A_4_^cpHMD^_pH1_) (Figure 9). In this case, the electrostatics’ interactions were poorly reproduced between the methods, exhibiting energy distributions in far ranges. The titratable amino acids of the A_4_D_2_A_4_ peptide were closer and, therefore, the interactions involving partial charges, which we remember were not correctly assigned in the backbone atoms, took a more relevant role in the electrostatics’ distributions. It can be appreciated in both the protonated and deprotonated (A_4_D_2_A_4_^cMD^_ASP_ and A_4_D_2_A_4_^cpHMD^_pH10_) simulations. Furthermore, the deprotonated form also showed mild deviations on the total energy, including angular, dihedral, or even the van der Waals energies (Figure 9). The dihedral and angular energies were not reproduced properly and, furthermore, the distribution of the Van der Waals interactions of A_4_D_2_A_4_^cpHMD^_pH10_ significantly disagreed with the rest of the simulations.

## 4. Conclusions

We extended the previous work of the titratable residues in the cpHMD method [36] by introducing explicit solvation in the simulations of the capped tripeptides. The Ramachandran maps or the energy distributions showed similar behaviours to the simulations with the implicit solvation model. Thus, we detected inconsistencies in conformational and energetical analyses of the deprotonated form of the cpHMD simulations due to the rough approach in the assignment of the partial charges of the backbone atoms, especially those amino acids with several protonation states. For these amino acids, we also observed minor shifts in electrostatics or conformational populations in the protonated form. However, in this case, we assumed that the distinct sampling on the protonation states between the methods could lead to these mismatches. In fact, the protonation state sampling of the cpHMD could suppose an advantage in simulations with Asp, Glu, or His amino acids. Parallelly, other minor artefacts may be involved in the reported deviations, such as correction of the partial charge of C_β_ atom or the ghost atoms during the simulation. It is worth to note that the dielectric constant of water was underestimated by the TIP3P water model, and simulations with other explicit water models could produce slightly different electrostatic profiles. However, since the effect of the discrepancy was because of the assignment of partial charges of backbone atoms, it was expected to remain independent of the water model.

After checking the strengths and weaknesses of the cpHMD method when using explicit solvent in the simulations, we studied the effect of the position of the titratable amino acids in a nonpolar chain in explicit solvent simulations. On one hand, the DA_8_D oligopeptide did not have remarkable deviations in the energy maps, clustering, and conformational properties of both protonated and deprotonated forms, a fact that indicates that structures with separated titratable amino acids can lead to similar conformational samplings. On the other hand, the simulations of A_4_D_2_A_4_ in the protonated form showed good agreement in the conformational and energy analyses when the cMD and cpHMD methods were compared, given that backbone partial charges were properly described. In contrast, the deprotonated form presented deviations in the measured properties (R_g_, f_SS_), the energy maps, and the clustering, suggesting that the approach taken in the partial charges of the deprotonated state significantly modified the conformational sampling and its interactions. Thus, to understand the limitations of the cpHMD methods, it would be convenient to have other properties related to the electrostatic environment of the systems, such as the interactions of titratable amino acids in sequences rich in polar or charged amino acids, the ionic strength, etc. Even so, this second study opens the door to seek strategies to minimize the limitations reported in the deprotonated form of the cpHMD method over the conformational sampling and, hopefully, carry out reliable simulations with this method in combination with experimental data.

## Figures and Tables

**Table 1:** Summary of the simulations indicating the peptide, the residue type, the simulation method, the protonation state (PS), and the intrinsic pKa of the amino acids. The PS labels indicate protonated (p), deprotonated (d), or titratable (t) residues while superscripts refer to positive (+), neutral (n), or negative (-) charge of the side chains. The PS of titratable residues depends on the pH conditions (1, 12, or 14) indicated below the cpHMD category.

Capped Tripeptides						
Residue	cMD	cpHMD			PS	Intrinsic pKa
		pH 1	pH 12	pH 14		
ASP	✓				d^-^	4.0
ASH	✓				p^n^	
AS4		✓	✓		t	
GLU	✓				d^-^	4.4
GLH	✓				p^n^	
GL4		✓	✓		t	
HIE	✓				d^n^	6.6
HID	✓				d^n^	
HIP	✓	✓	✓		p^+^/t	
CYM	✓				d-	8.5
CYS	✓	✓	✓		p^n^/t	
TYR	✓	✓			p^n^/t	9.6
LYN	✓				d^n^	10.4
LYS	✓	✓		✓	p^+^/t	
DA_8_D						
		**pH 1**	**pH 10**			
ASP	✓				d^-^	4.0
ASH	✓				p^n^	
AS4		✓	✓		t	
A_4_D_2_A_4_						
		**pH 1**	**pH 10**			
ASP	✓				d^-^	4.0
ASH	✓				p^n^	
AS4		✓	✓		t	

## Data Availability

The data presented in this study are available on request from the corresponding author.

## Supplementary Materials

The following are available online at https://www.mdpi.com/article/10.3390/polym13193311/s1. Figure S1: Classification of the nine secondary structure regions (C5, PII, αD, β2, C7eq, αL, α’, αR and C7axial) in the Ramachandran space by J. Rubio-Martinez et al. Figure S2: Ramachandran maps of the capped Glu2 tripeptide. Titles indicate the residues, the simulation method in the superscripts and the pH value in the subscripts. Each simulation conditions have two energy maps according to the set of backbone dihedral angles of the N-terminal (φ1/ψ1) or the C-terminal amino acid (φ2/ψ2). Solid lines indicate an increase of 0.6 kcal/mol in the energy map. Figure S3: Populations of the conformational regions (PII, αR, C7eq, C5 and αL) in the Ramachandran maps of each amino acid of the capped Glu2 tripeptide. Labels indicate the residues, the simulation method in the superscripts and the pH value in the subscripts. Net charge of the tripeptide is below (q). Striped or solid box style are protonated or deprotonated states, respectively. Figure S4: Ramachandran maps of the capped His2 tripeptide. Titles indicate the residues, the simulation method in the superscripts and the pH value in the subscripts. Each simulation conditions have two energy maps according to the set of backbone dihedral angles of the N-terminal (φ1/ψ1) or the C-terminal amino acid (φ2/ψ2). Solid lines indicate an increase of 0.6 kcal/mol in the energy map. Figure S5: Populations of the conformational regions (PII, αR, C7eq, C5 and αL) in the Ramachandran maps of each amino acid of the capped His2 tripeptide. Labels indicate the residues, the simulation method in the superscripts and the pH value in the subscripts. Net charge of the tripeptide is below (q). Striped or solid box style are protonated or deprotonated states, respectively. Figure S6: Ramachandran maps of the capped Lys2 tripeptide. Titles indicate the residues, the simulation method in the superscripts and the pH value in the subscripts. Each simulation conditions have two energy maps according to the set of backbone dihedral angles of the N-terminal (φ1/ψ1) or the C-terminal amino acid (φ2/ψ2). Solid lines indicate an increase of 0.6 kcal/mol in the energy map. Figure S7: Ramachandran maps of the capped Tyr2 tripeptide. Titles indicate the residues, the simulation method in the superscripts and the pH value in the subscripts. Each simulation conditions have two energy maps according to the set of backbone dihedral angles of the N-terminal (φ1/ψ1) or the C-terminal amino acid (φ2/ψ2). Solid lines indicate an increase of 0.6 kcal/mol in the energy map. Figure S8: Ramachandran maps of the capped Cys2 tripeptide. Titles indicate the residues, the simulation method in the superscripts and the pH value in the subscripts. Each simulation conditions have two energy maps according to the set of backbone dihedral angles of the N-terminal (φ1/ψ1) or the C-terminal amino acid (φ2/ψ2). Solid lines indicate an increase of 0.6 kcal/mol in the energy map. Figure S9: Populations of the conformational regions (PII, αR, C7eq, C5 and αL) in the Ramachandran maps of each amino acid of the capped Lys2 tripeptide. Labels indicate the residues, the simulation method in the superscripts and the pH value in the subscripts. Net charge of the tripeptide is below (q). Striped or solid box style are protonated or deprotonated states, respectively. Figure S10: Populations of the conformational regions (PII, αR, C7eq, C5 and αL) in the Ramachandran maps of each amino acid of the capped Tyr2 tripeptide. Labels indicate the residues, the simulation method in the superscripts and the pH value in the subscripts. Net charge of the tripeptide is below (q). Striped or solid box style are protonated or deprotonated states, respectively. Figure S11: Populations of the conformational regions (PII, αR, C7eq, C5 and αL) in the Ramachandran maps of each amino acid of the capped Cys2 tripeptide. Labels indicate the residues, the simulation method in the superscripts and the pH value in the subscripts. Net charge of the tripeptide is below (q). Striped or solid box style are protonated or deprotonated states, respectively. Figure S12: Energy distributions of the capped Asp2 tripeptide without solvent molecules. Dotted and dashed lines are cpHMD and cMD simulations, respectively. Figure S13: Energy distribution of the 1–4 and long-range electrostatics capped Asp2 tripeptide divided in backbone and side chain atoms. Dotted and dashed lines are cpHMD and cMD simulation methods, respectively. Figure S14: Energy distributions of the capped Glu2 tripeptide without solvent molecules. Dotted and dashed lines are cpHMD and cMD simulations, respectively. Figure S15: Energy distribution of the 1–4 and long-range electrostatics capped Glu2 tripeptide divided in backbone and side chain atoms. Dotted and dashed lines are cpHMD and cMD simulation methods, respectively. Figure S16: Energy distributions of the capped His2 tripeptide without solvent molecules. Dotted and dashed lines are cpHMD and cMD simulations, respectively. “HIPcpHMDpH12 δ” and “HIPcpHMDpH12 ε” are the energy distributions calculated using partial charges fixed on the δ and ε protonation state. Figure S17: Energy distribution of the 1-4 and long-range electrostatics capped His2 tripeptide divided in backbone and side chain atoms. Dotted and dashed lines are cpHMD and cMD simulation methods, respectively. “HIPcpHMDpH12 δ” and “HIPcpHMDpH12 ε” are the energy distributions calculated using partial charges fixed on the δ and ε protonation state. Figure S18: Energy distributions of the capped Lys2 tripeptide without solvent molecules. Dotted and dashed lines are cpHMD and cMD simulations, respectively. Figure S19: Energy distributions of the capped Tyr2 tripeptide without solvent molecules. Dotted and dashed lines are cpHMD and cMD simulations, respectively. Figure S20: Energy distributions of the capped Cys2 tripeptide without solvent molecules. Dotted and dashed lines are cpHMD and cMD simulations, respectively. Figure S21: Energy distribution of the 1–4 and long-range electrostatics capped Lys2 tripeptide divided in backbone and side chain atoms. Dotted and dashed lines are cpHMD and cMD simulation methods, respectively. Figure S22: Energy distribution of the 1-4 and long-range electrostatics capped Tyr2 tripeptide divided in backbone and side chain atoms. Dotted and dashed lines are cpHMD and cMD simulation methods, respectively. Figure S23: Energy distribution of the 1-4 and long-range electrostatics capped Cys2 tripeptide divided in backbone and side chain atoms. Dotted and dashed lines are cpHMD and cMD simulation methods, respectively. Figure S24: 2D-RMSD of the six first representative conformations of DA8D peptide in protonated form (DA8DcMDASH and DA8DcpHMDpH1). RMSD is calculated using the Cα atoms of the peptides. Figure S25: 2D-RMSD of the six first representative conformations of DA8D peptide in the deprotonated form (DA8DcMDASP and DA8DcpHMDpH10). RMSD is calculated using the Cα atoms of the peptides. Figure S26: Distribution of the three first PC at several simulation times (2, 4, 8 and 10 µs) of the DA8D peptide. Deprotonated and protonated form are in the left and right, respectively. Dotted and dashed lines are cpHMD and cMD simulation methods, respectively. Figure S27: 2D-RMSD of the six first representative conformations of A4D2A4 peptide in protonated form (A4D2A4cMDASH and A4D2A4cpHMDpH1). RMSD is calculated using the Cα atoms of the peptides. Figure S28: 2D-RMSD of the six first representative conformations of A4D2A4 peptide in the deprotonated form (A4D2A4cMDASP and A4D2A4cpHMDpH10). RMSD is calculated using the Cα atoms of the peptides.

## References

[1] Nerenberg P.S., Head-Gordon T. (2018). New developments in force fields for biomolecular simulations. Curr. Opin. Struct. Biol..

[2] Sugita Y., Okamoto Y. (1999). Replica-exchange molecular dynamics method for protein folding. Chem. Phys. Lett..

[3] Laio A., Parrinello M. (2002). Escaping free-energy minima. Proc. Natl. Acad. Sci. USA.

[4] Hamelberg D., Mongan J., Mccammon J.A. (2004). Accelerated molecular dynamics: A promising and efficient simulation method for biomolecules. J. Chem. Phys..

[5] Harris R.C., Tsai C.-C., Ellis C.R., Shen J. (2017). Proton-Coupled Conformational Allostery Modulates the Inhibitor Selectivity for β-Secretase. J. Phys. Chem. Lett..

[6] Radak B.K., Chipot C., Suh D., Jo S., Jiang W., Phillips J.C., Schulten K., Roux B. (2017). Constant-pH Molecular Dynamics Simulations for Large Biomolecular Systems. J. Chem. Theory Comput..

[7] Huang Y., Yue Z., Tsai C.-C., Henderson J.A., Shen J. (2018). Predicting Catalytic Proton Donors and Nucleophiles in Enzymes: How Adding Dynamics Helps Elucidate the Structure−Function Relationships. J. Phys. Chem. Lett..

[8] Hofer F., Kraml J., Kahler U., Kamenik A.S., Liedl K.R. (2020). Catalytic Site pK a Values of Aspartic, Cysteine, and Serine Proteases: Constant pH MD Simulations. J. Chem. Inf. Model..

[9] Reis P.B.P.S., Vila-Viç D., Rocchia W., Machuqueiro M. (2020). PypKa: A Flexible Python Module for Poisson—Boltzmann-Based p*K*_a_ Calculations. J. Chem. Inf. Model..

[10] Anandakrishnan R., Aguilar B., Onufriev A.V. (2012). H+ + 3.0: Automating pK prediction and the preparation of biomolecular structures for atomistic molecular modeling and simulations. Nucleic Acids Res..

[11] Olsson M.H.M., Søndergaard C.R., Rostkowski M., Jensen J.H. (2011). PROPKA3: Consistent Treatment of Internal and Surface Residues in Empirical pK a Predictions. J. Chem. Theory Comput..

[12] Barazorda-Ccahuana H.L., Gómez B., Mas F., Madurga S. (2020). Effect of pH on the Supramolecular Structure of Helicobacter pylori Urease by Molecular Dynamics Simulations. Polymers.

[13] Beroza P., Fredkin D.R., Okamura M.Y., Feher G. (1991). Protonation of interacting residues in a protein by a Monte Carlo method: Application to lysozyme and the photosynthetic reaction center of Rhodobacter sphaeroides. Proc. Natl. Acad. Sci. USA.

[14] Mertz J.E., Pettitt B.M. (1994). Molecular Dynamics At a Constant pH. Int. J. High Perform. Comput. Appl..

[15] Baptista A.M., Martel P.J., Petersen S.B. (1997). Simulation of protein conformational freedom as a function of pH: Constant-pH molecular dynamics using implicit titration. Proteins Struct. Funct. Bioinform..

[16] Lee M.S., Salsbury F.R., Brooks C.L. (2004). Constant-pH Molecular Dynamics Using Continuous Titration Coordinates. Proteins Struct. Funct. Bioinform..

[17] Donnini S., Tegeler F., Groenhof G., Grubmüller H. (2011). Constant pH Molecular Dynamics in Explicit Solvent with λ-Dynamics. J. Chem. Theory Comput..

[18] Wallace J.A., Shen J.K. (2011). Continuous Constant pH Molecular Dynamics in Explicit Solvent with pH-Based Replica Exchange. J. Chem. Theory Comput..

[19] Goh G.B., Hulbert B.S., Zhou H., Brooks III C.L. (2014). Constant pH Molecular Dynamics of Proteins in Explicit Solvent with Proton Tautomerism. Proteins.

[20] Baptista A.M., Teixeira V.H., Soares C.M., Nio A., Baptista M., Udio C., Soares M. (2002). Constant-pH molecular dynamics using stochastic titration. J. Chem. Phys..

[21] Bürgi R., Kollman P.A., Van Gunsteren W.F. (2002). Simulating proteins at constant pH: An approach combining molecular dynamics and Monte Carlo simulation. Proteins Struct. Funct. Genet..

[22] Mongan J., Case D.A., McCammon J.A. (2004). Constant pH molecular dynamics in generalized Born implicit solvent. J. Comput. Chem..

[23] Swails J.M., York D.M., Roitberg A.E. (2014). Constant pH Replica Exchange Molecular Dynamics in Explicit Solvent Using Discrete Protonation States: Implementation, Testing, and Validation. J. Chem. Theory Comput..

[24] Mongan J., Case D.A. (2005). Biomolecular simulations at constant pH. Curr. Opin. Struct. Biol..

[25] Williams S.L., Blachly P.G., Mccammon J.A. (2011). Measuring the successes and deficiencies of constant pH molecular dynamics: A blind prediction study. Proteins Struct. Funct. Bioinform..

[26] Machuqueiro M., Baptista A.M. (2011). Is the prediction of pKa values by constant-pH molecular dynamics being hindered by inherited problems?. Proteins Struct. Funct. Bioinform..

[27] Wallace J.A., Wang Y., Shi C., Pastoor K.J., Nguyen B.-L., Xia K., Shen J.K. (2011). Toward accurate prediction of pKa values for internal protein residues: The importance of conformational relaxation and desolvation energy. Proteins Struct. Funct. Bioinform..

[28] Chen W., Morrow B.H., Shi C., Shen J.K. (2014). Recent development and application of constant pH molecular dynamics. Mol. Simul..

[29] Dobrev P., Phani S., Vemulapalli B., Nath N., Griesinger C., Grubmü H. (2020). Probing the Accuracy of Explicit Solvent Constant pH Molecular Dynamics Simulations for Peptides. J. Chem. Theory Comput..

[30] Itoh S.G., Damjanović A., Brooks B.R. (2011). pH replica-exchange method based on discrete protonation states. Proteins Struct. Funct. Bioinform..

[31] Machuqueiro M., Baptista A.M. (2006). Constant-pH Molecular Dynamics with Ionic Strength Effects: Protonation-Conformation Coupling in Decalysine. J. Phys. Chem. B.

[32] Williams S.L., De Oliveira C.A.F., Mccammon J.A. (2010). Coupling Constant pH Molecular Dynamics with Accelerated Molecular Dynamics. J. Chem. Theory Comput..

[33] Chen W., Wallace J.A., Yue Z., Shen J.K. (2013). Introducing Titratable Water to All-Atom Molecular Dynamics at Constant pH. Biophys. J..

[34] Yeager A.V., Swails J.M., Miller B.R. (2017). Improved Accuracy for Constant pH-REMD Simulations through Modification of Carboxylate Effective Radii. J. Chem. Theory Comput..

[35] Case D.A., Cheatham T.E., Darden T., Gohlke H., Luo R., Merz K.M., Onufriev A., Simmerling C., Wang B., Woods R.J. (2005). The Amber biomolecular simulation programs. J. Comput. Chem..

[36] Privat C., Madurga S., Mas F., Rubio-Martínez J. (2021). On the Use of the Discrete Constant pH Molecular Dynamics to Describe the Conformational Space of Peptides. Polymers.

[37] Jorgensen W.L., Chandrasekhar J., Madura J.D., Impey R.W., Klein M.L. (1983). Comparison of simple potential functions for simulating liquid water. J. Chem. Phys..

[38] Onufriev A., Bashford D., Case D.A. (2000). Modification of the Generalized Born Model Suitable for Macromolecules. J. Phys. Chem. B.

[39] Perez J.J., Santos Tomas M., Rubio-Martinez J. (2016). Assessment of the Sampling Performance of Multiple-Copy Dynamics versus a Unique Trajectory. J. Chem. Inf. Model..

