# Supplementary material for: Unravelling Constant pH Molecular Dynamics in Oligopeptides with Explicit Solvation Model
Source: Polymers (Basel). 2021 Sep 28;13(19):3311. doi: 10.3390/polym13193311 (PMC8512540; doi:10.3390/polym13193311)
Supplement: Supplementary file 1 [file polymers-13-03311-s001.zip › polymers-1394672-supplementary.pdf]

Unravelling Constant pH Molecular Dynamics in Oligopeptides with Explicit Solvation Model

Cristian Privat, Sergio Madurga *, Francesc Mas and Jaime Rubio-Martinez *

Department of Material Science and Physical Chemistry & Research Institute of Theoretical and Computational Chemistry (IQTCUB), University of Barcelona, C/Martí i Franquès 1, 08028 Barcelona, Spain; cprivat@ub.edu (C.P.); fmas@ub.edu (F.M.)
* Correspondence: s.madurga@ub.edu (S.M.); jaime.rubio@ub.edu (J.R.-M.)

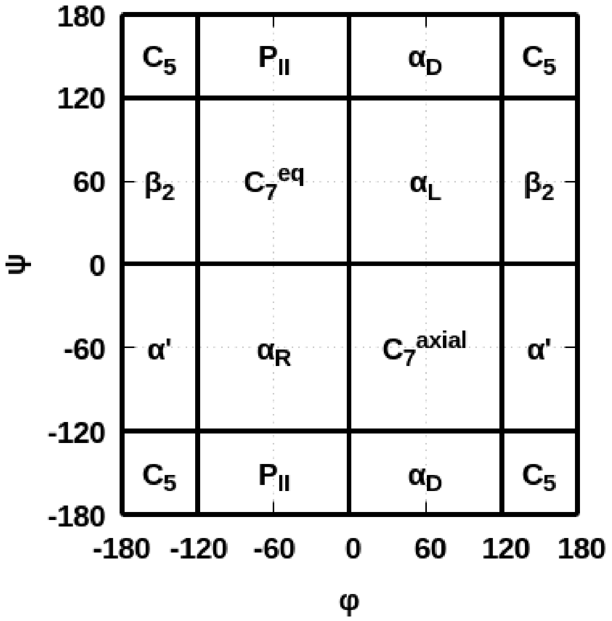

Figure S1. Classification of the nine secondary structure regions (C_5, P_{II}, α_D, β_2, C_7^{eq}, α_L, α', α_R and C_7^{axial}) in the Ramachandran space by J. Rubio-Martinez et al. [1].

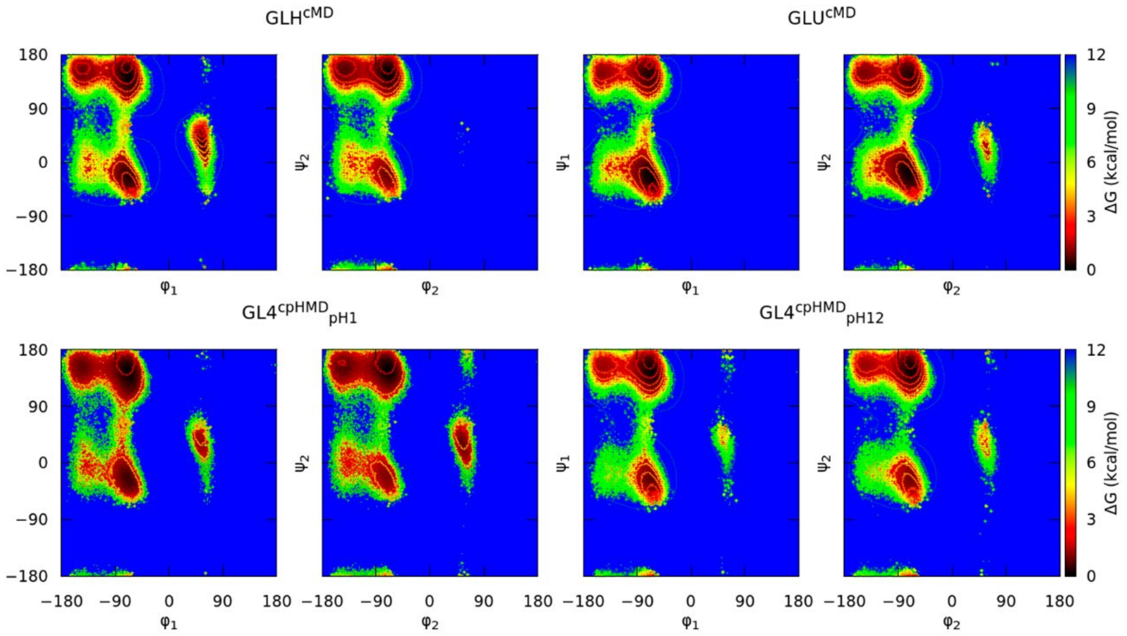

Figure S2. Ramachandran maps of the capped Glu_2 tripeptide. Titles indicate the residues, the simulation method in the superscripts and the pH value in the subscripts. Each simulation conditions have two energy maps according to the set of backbone dihedral angles of the N-terminal (φ_1/ψ_1) or the C-terminal amino acid (φ_2/ψ_2). Solid lines indicate an increase of 0.6 kcal/mol in the energy map.

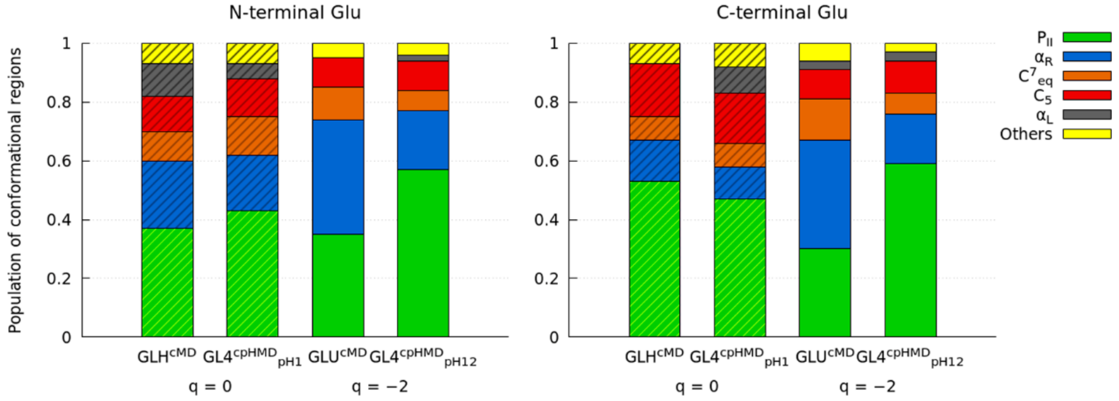

Figure S3. Populations of the conformational regions (P_{II}, α_R, C^7_{eq}, C_5 and α_L) in the Ramachandran maps of each amino acid of the capped Glu_2 tripeptide. Labels indicate the residues, the simulation method in the superscripts and the pH value in the subscripts. Net charge of the tripeptide is below (q). Striped or solid box style are protonated or deprotonated states, respectively.

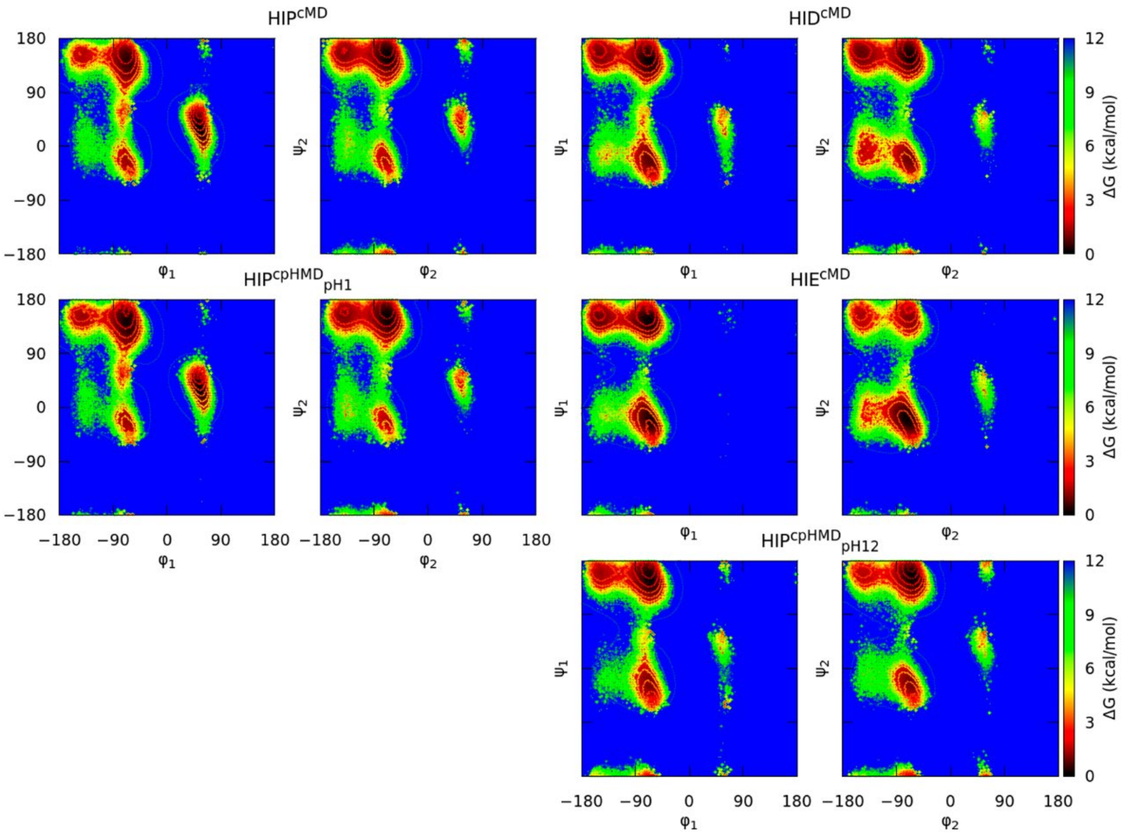

Figure S4. Ramachandran maps of the capped His_2 tripeptide. Titles indicate the residues, the simulation method in the superscripts and the pH value in the subscripts. Each simulation conditions have two energy maps according to the set of backbone dihedral angles of the N-terminal (φ_1/ψ_1) or the C-terminal amino acid (φ_2/ψ_2). Solid lines indicate an increase of 0.6 kcal/mol in the energy map.

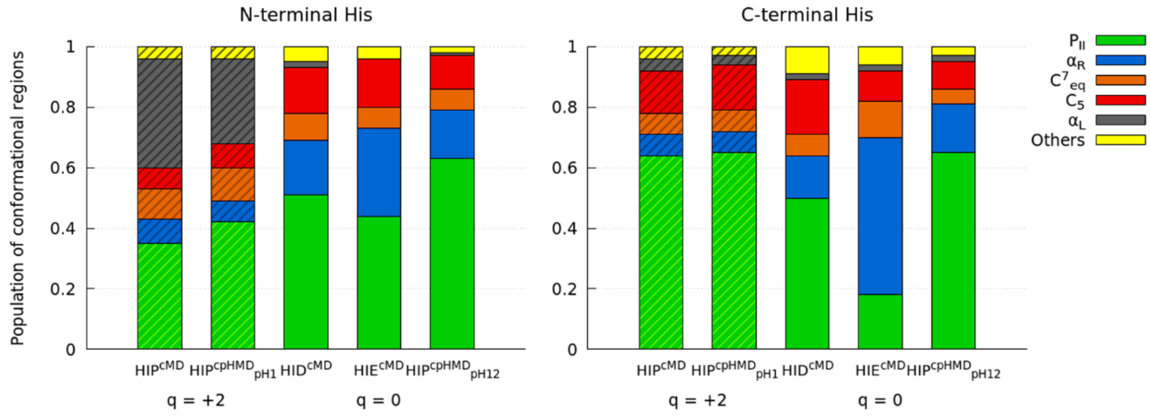

Figure S5. Populations of the conformational regions (P_{II}, α_R, C^7_{eq}, C_5 and α_L) in the Ramachandran maps of each amino acid of the capped His_2 tripeptide. Labels indicate the residues, the simulation method in the superscripts and the pH value in the subscripts. Net charge of the tripeptide is below (q). Striped or solid box style are protonated or deprotonated states, respectively.

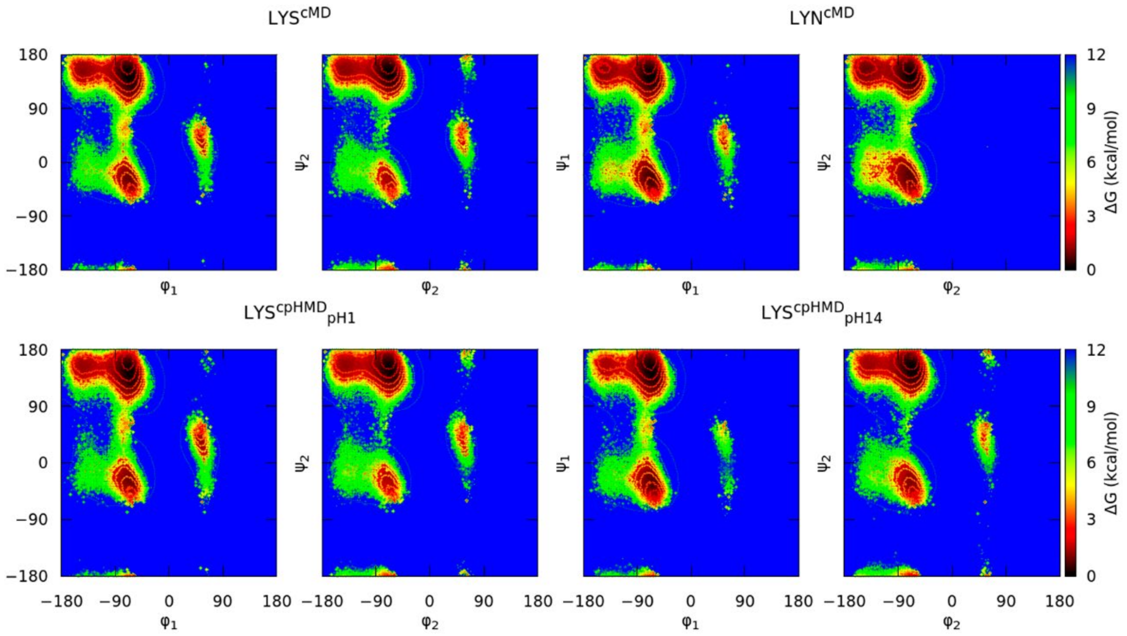

Figure S6. Ramachandran maps of the capped Lys_2 tripeptide. Titles indicate the residues, the simulation method in the superscripts and the pH value in the subscripts. Each simulation conditions have two energy maps according to the set of backbone dihedral angles of the N-terminal (φ_1/ψ_1) or the C-terminal amino acid (φ_2/ψ_2). Solid lines indicate an increase of 0.6 kcal/mol in the energy map.

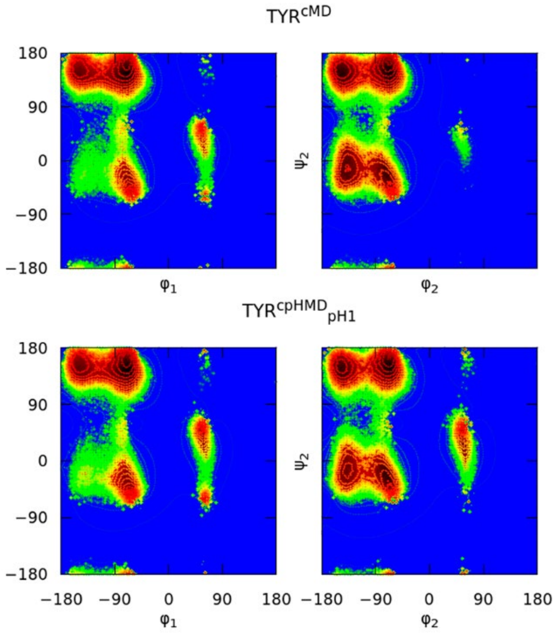

Figure S7. Ramachandran maps of the capped Tyr_2 tripeptide. Titles indicate the residues, the simulation method in the superscripts and the pH value in the subscripts. Each simulation conditions have two energy maps according to the set of backbone dihedral angles of the N-terminal (φ_1/ψ_1) or the C-terminal amino acid (φ_2/ψ_2). Solid lines indicate an increase of 0.6 kcal/mol in the energy map.

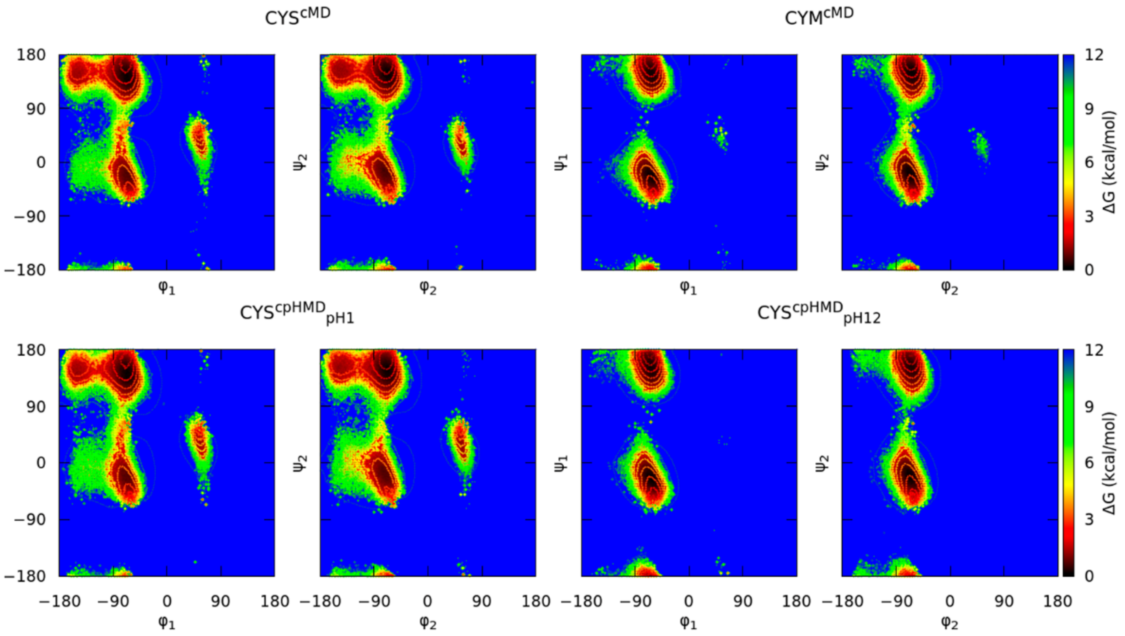

Figure S8. Ramachandran maps of the capped Cys_2 tripeptide. Titles indicate the residues, the simulation method in the superscripts and the pH value in the subscripts. Each simulation conditions have two energy maps according to the set of backbone dihedral angles of the N-terminal (φ_1/ψ_1) or the C-terminal amino acid (φ_2/ψ_2). Solid lines indicate an increase of 0.6 kcal/mol in the energy map.

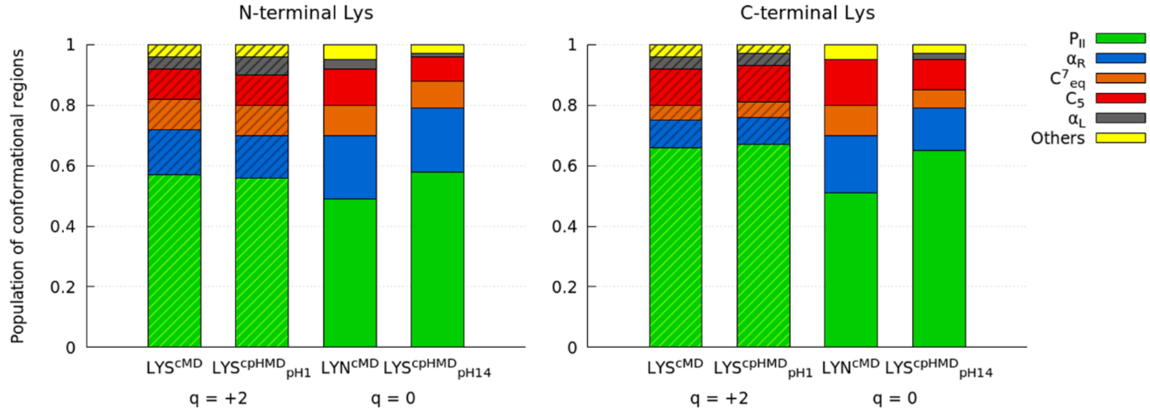

Figure S9. Populations of the conformational regions (P_{II}, α_R, C^7_{eq}, C_5 and α_L) in the Ramachandran maps of each amino acid of the capped Lys_2 tripeptide. Labels indicate the residues, the simulation method in the superscripts and the pH value in the subscripts. Net charge of the tripeptide is below (q). Striped or solid box style are protonated or deprotonated states, respectively.

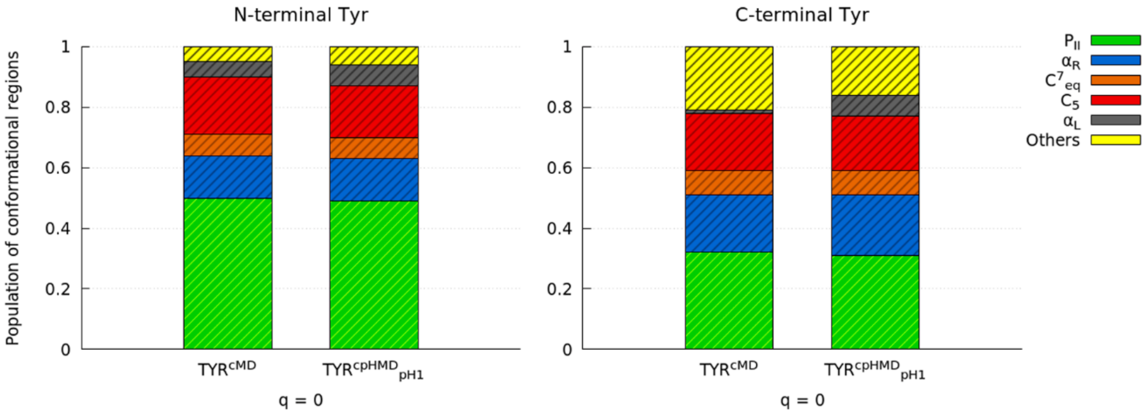

Figure S10. Populations of the conformational regions (P_{II}, α_R, C^7_{eq}, C_5 and α_L) in the Ramachandran maps of each amino acid of the capped Tyr_2 tripeptide. Labels indicate the residues, the simulation method in the superscripts and the pH value in the subscripts. Net charge of the tripeptide is below (q). Striped or solid box style are protonated or deprotonated states, respectively.

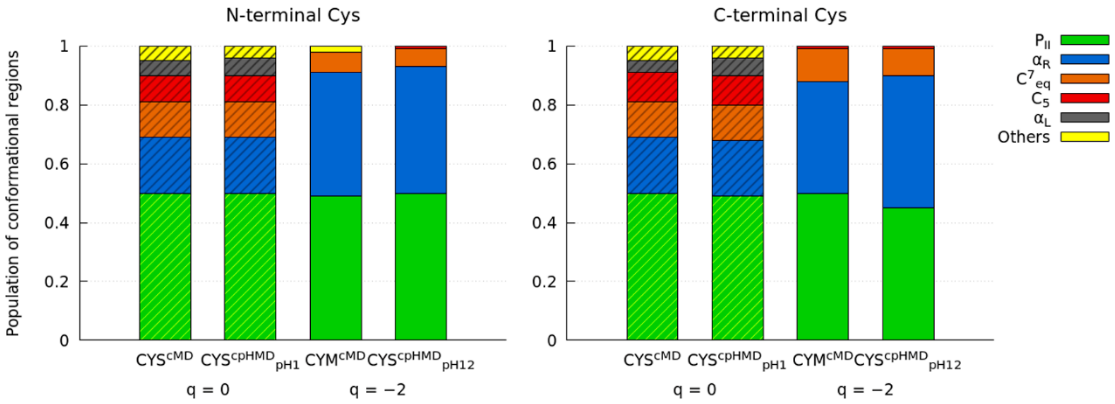

Figure S11. Populations of the conformational regions (P_{II}, α_R, C^7_{eq}, C_5 and α_L) in the Ramachandran maps of each amino acid of the capped Cys_2 tripeptide. Labels indicate the residues, the simulation method in the superscripts and the pH value in the subscripts. Net charge of the tripeptide is below (q). Striped or solid box style are protonated or deprotonated states, respectively.

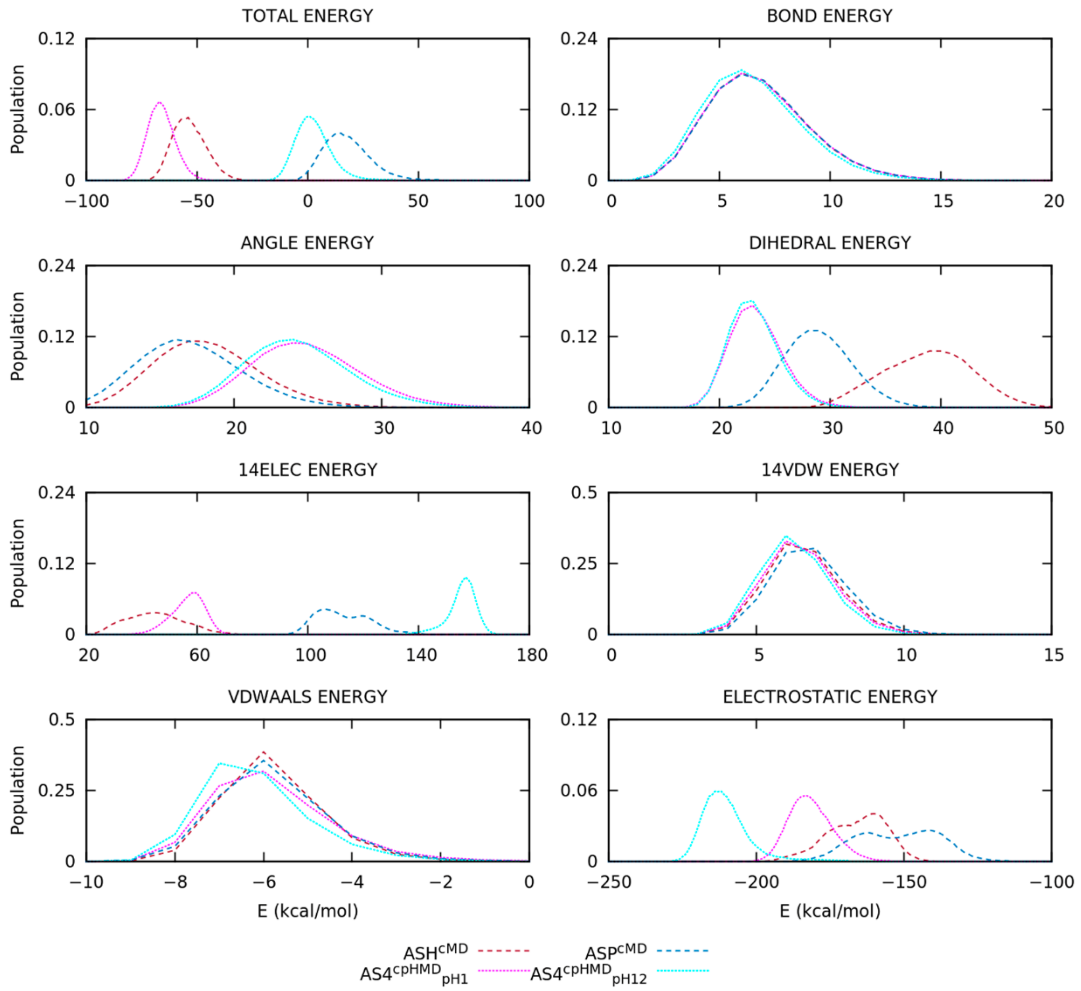

Figure S12. Energy distributions of the capped Asp_2 tripeptide without solvent molecules. Dotted and dashed lines are cpHMD and cMD simulations, respectively.

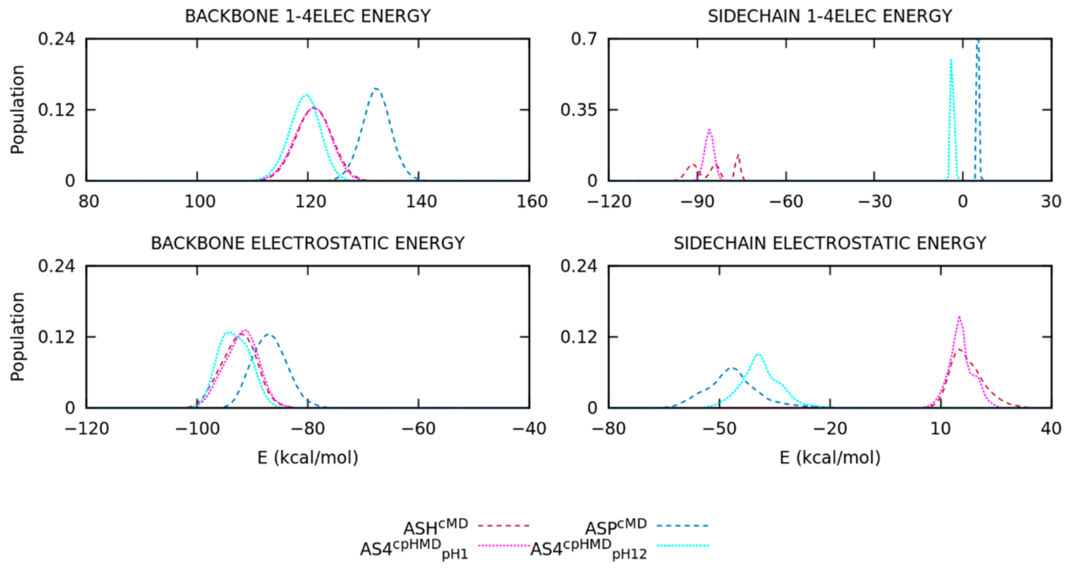

Figure S13. Energy distribution of the 1-4 and long-range electrostatics capped Asp2 tripeptide divided in backbone and side chain atoms. Dotted and dashed lines are cpHMD and cMD simulation methods, respectively.

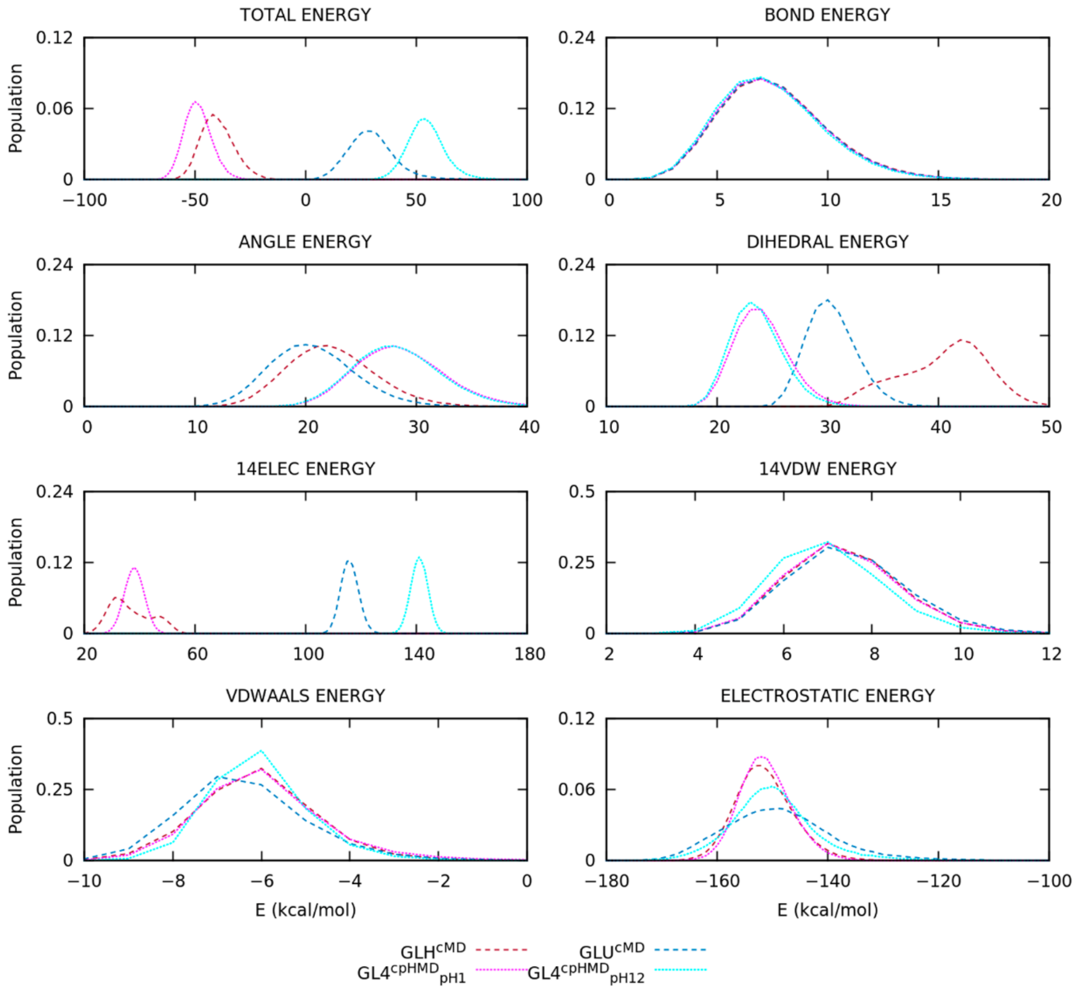

Figure S14. Energy distributions of the capped Glu2 tripeptide without solvent molecules. Dotted and dashed lines are cpHMD and cMD simulations, respectively.

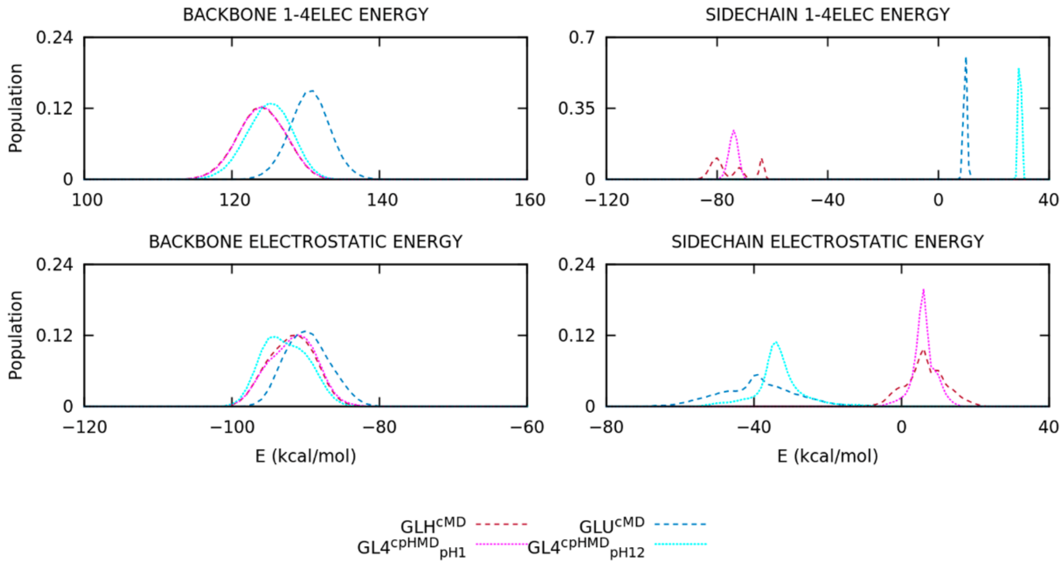

Figure S15. Energy distribution of the 1-4 and long-range electrostatics capped Glu_2 tripeptide divided in backbone and side chain atoms. Dotted and dashed lines are cpHMD and cMD simulation methods, respectively.

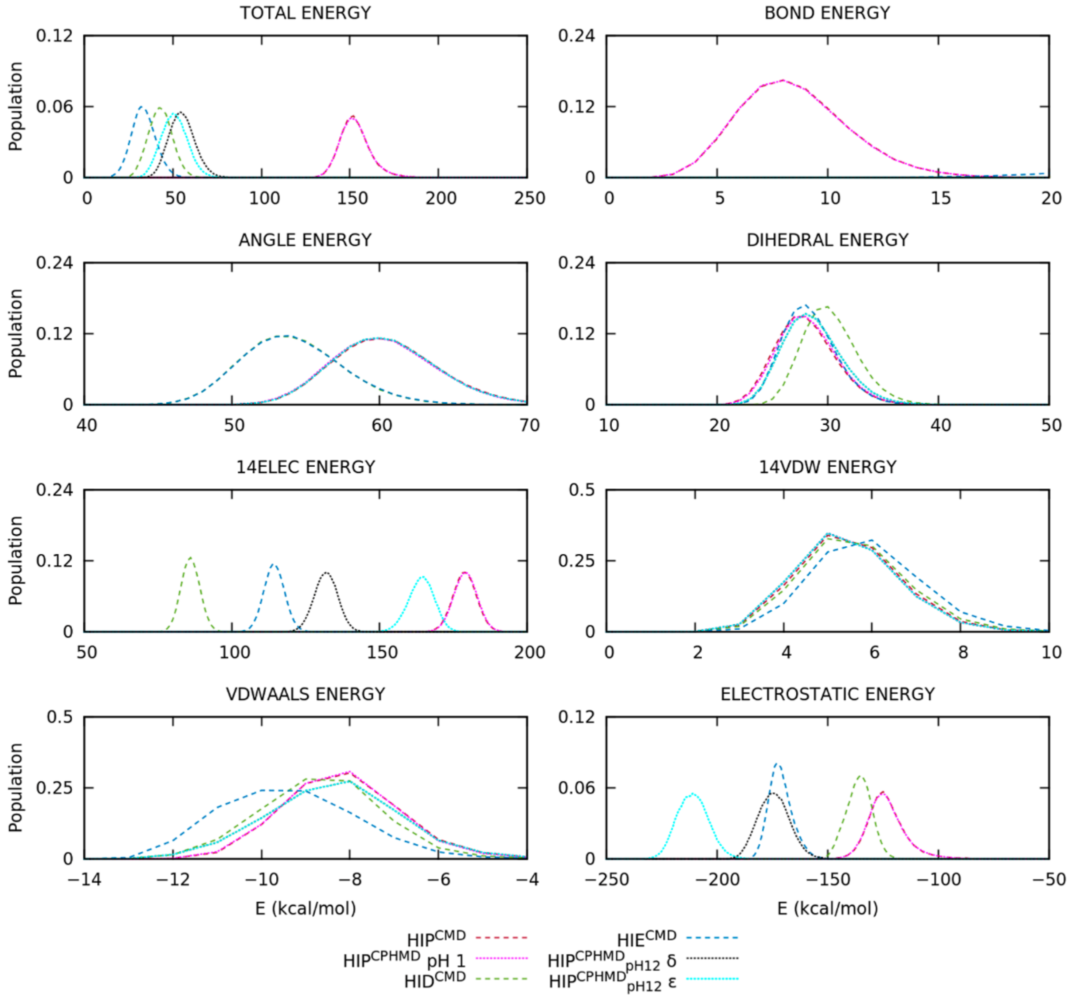

Figure S16. Energy distributions of the capped His_2 tripeptide without solvent molecules. Dotted and dashed lines are cpHMD and cMD simulations, respectively. “$HIP^{cpHMD}{}_{pH12}$ δ” and “$HIP^{cpHMD}{}_{pH12}$ ε” are the energy distributions calculated using partial charges fixed on the δ and ε protonation state.

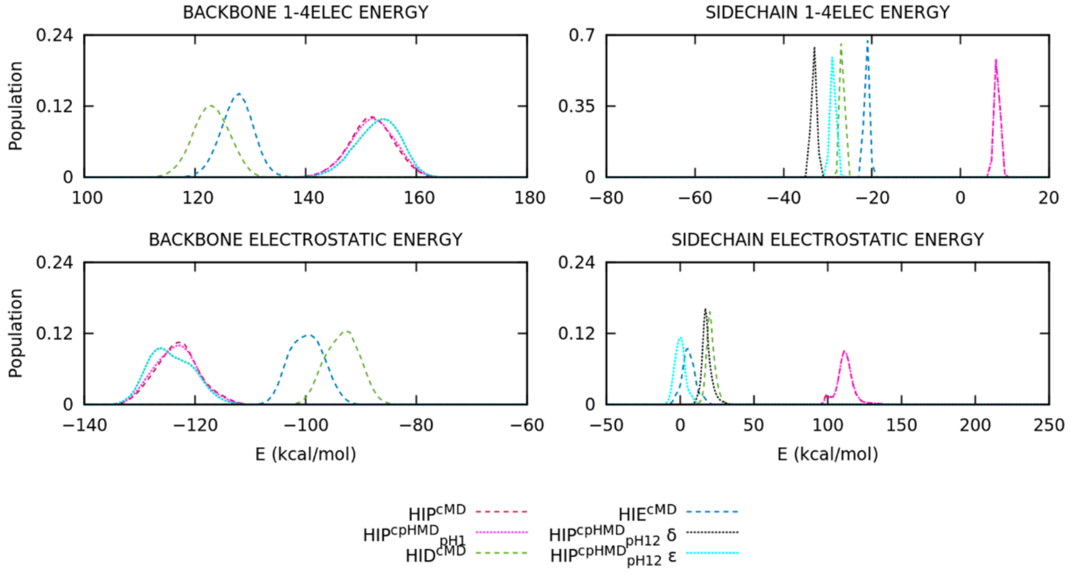

Figure S17. Energy distribution of the 1-4 and long-range electrostatics capped His_2 tripeptide divided in backbone and side chain atoms. Dotted and dashed lines are cpHMD and cMD simulation methods, respectively. "$HIP^{cpHMD}{}_{pH12}$ δ" and "$HIP^{cpHMD}{}_{pH12}$ ε" are the energy distributions calculated using partial charges fixed on the δ and ε protonation state.

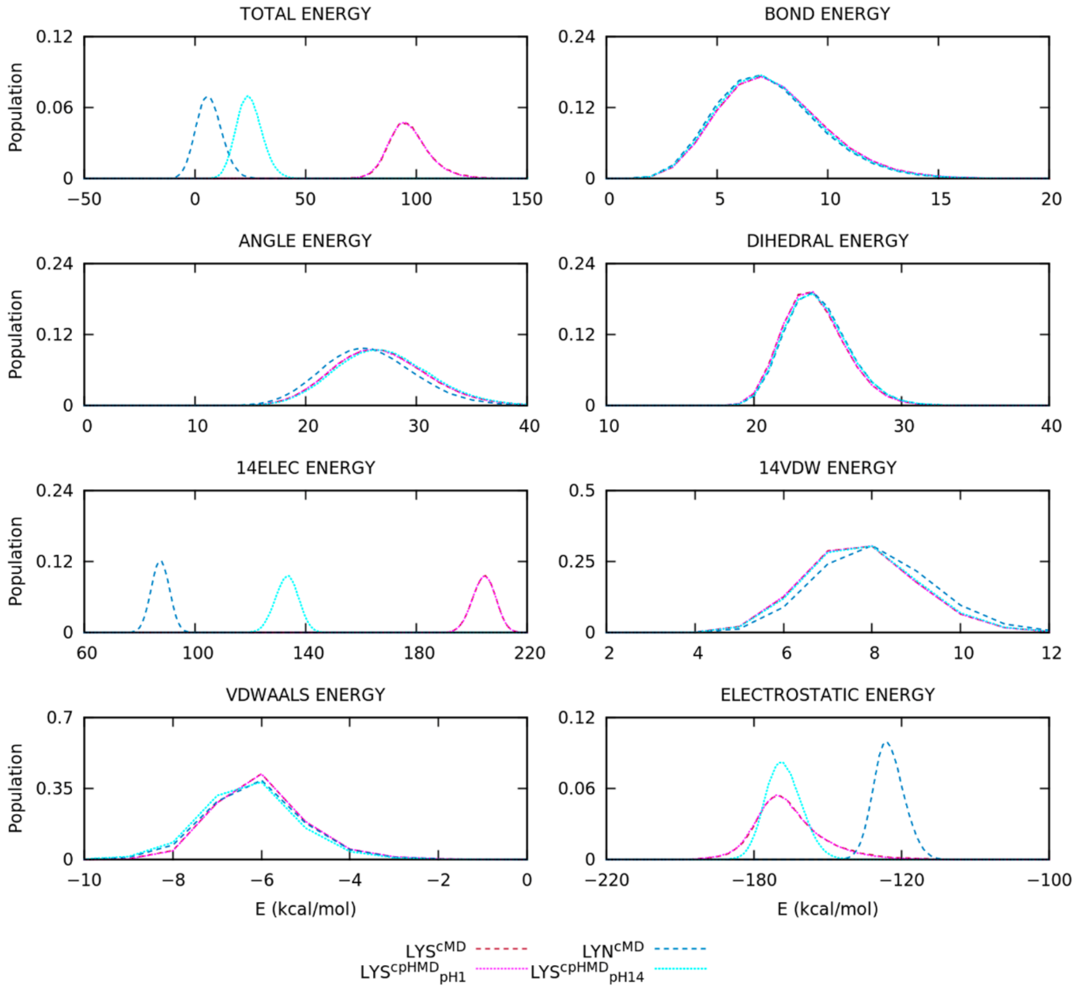

Figure S18. Energy distributions of the capped Lys$_2$ tripeptide without solvent molecules. Dotted and dashed lines are cpHMD and cMD simulations, respectively.

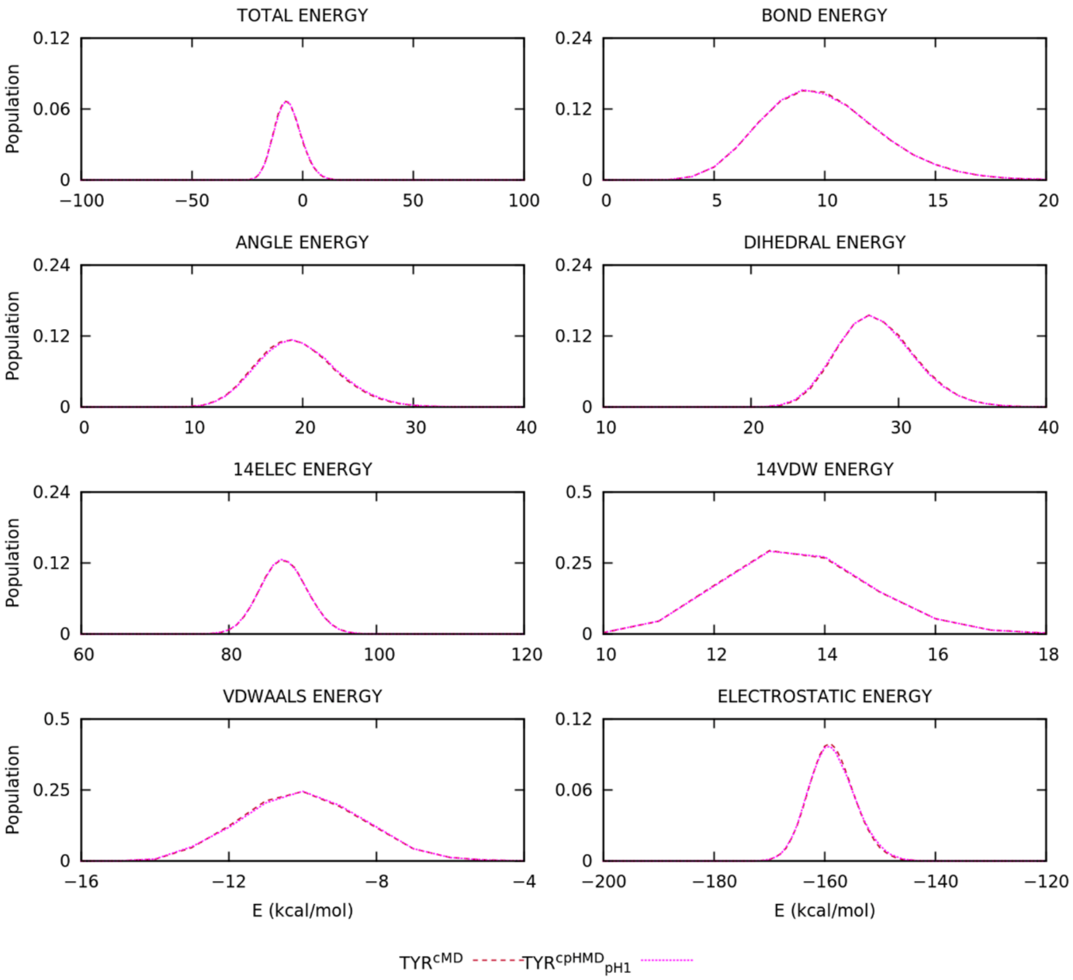

Figure S19. Energy distributions of the capped Tyr$_2$ tripeptide without solvent molecules. Dotted and dashed lines are cpHMD and cMD simulations, respectively.

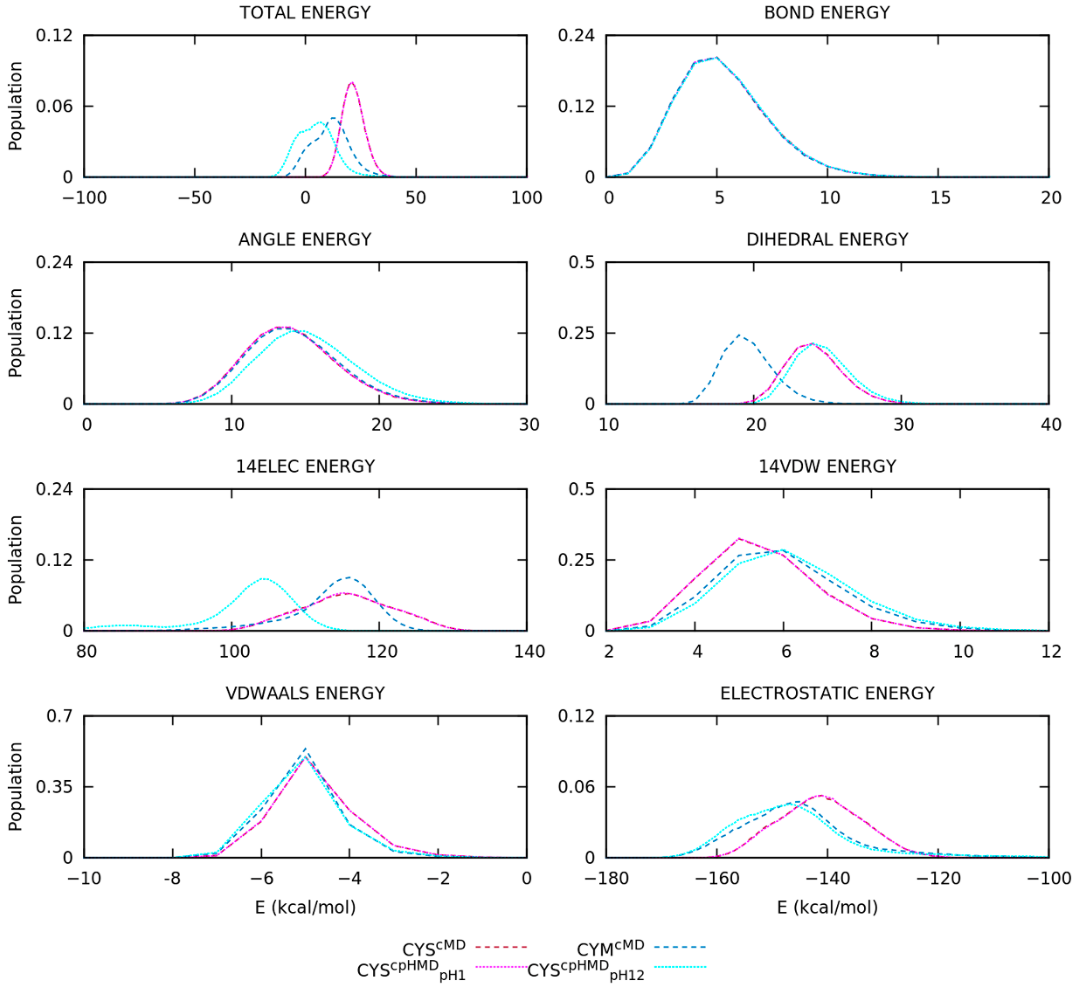

Figure S20. Energy distributions of the capped Cys_2 tripeptide without solvent molecules. Dotted and dashed lines are cpHMD and cMD simulations, respectively.

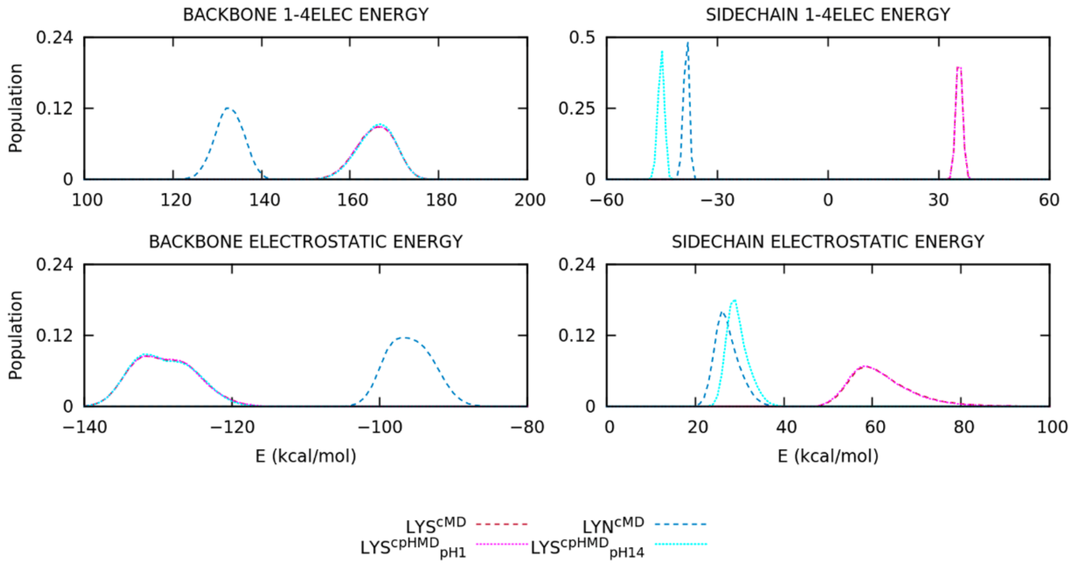

Figure S21. Energy distribution of the 1-4 and long-range electrostatics capped Lys2 tripeptide divided in backbone and side chain atoms. Dotted and dashed lines are cpHMD and cMD simulation methods, respectively.

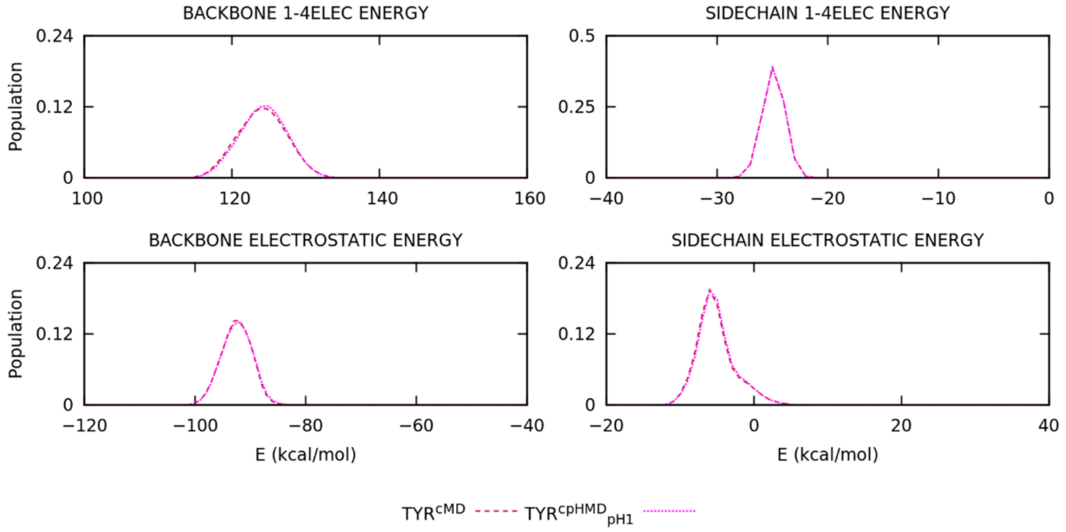

Figure S22. Energy distribution of the 1-4 and long-range electrostatics capped Tyr2 tripeptide divided in backbone and side chain atoms. Dotted and dashed lines are cpHMD and cMD simulation methods, respectively.

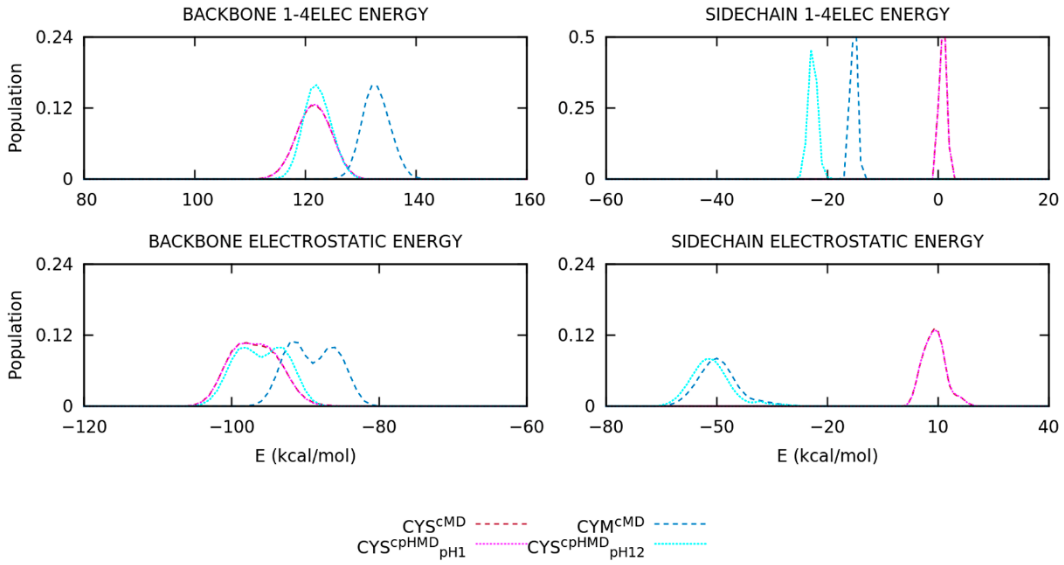

Figure S23. Energy distribution of the 1-4 and long-range electrostatics capped Cys_2 tripeptide divided in backbone and side chain atoms. Dotted and dashed lines are cpHMD and cMD simulation methods, respectively.

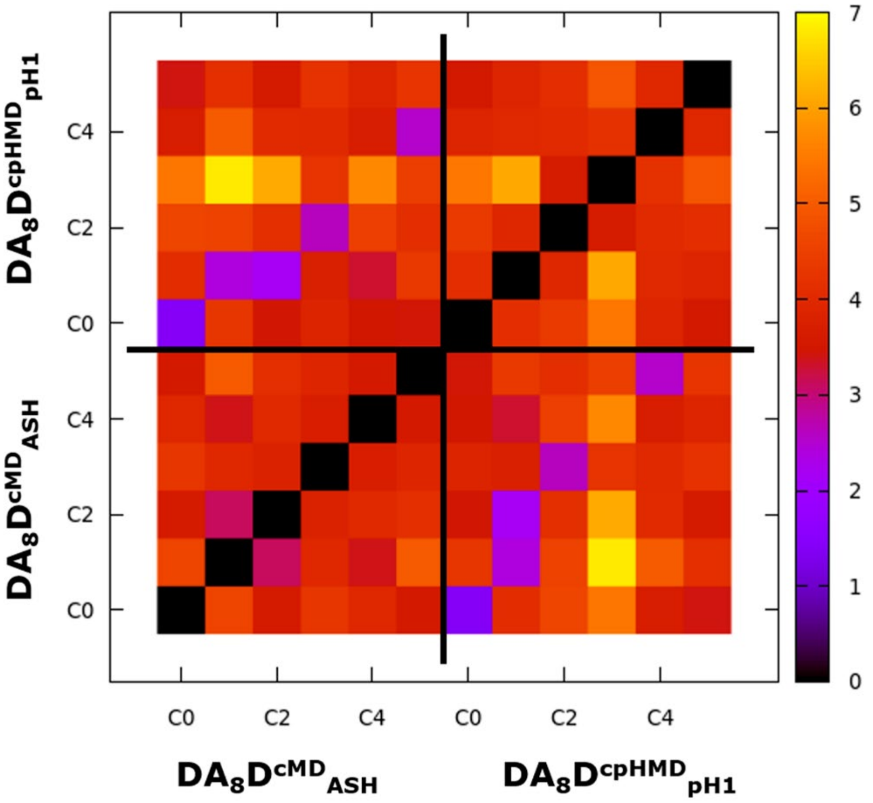

Figure S24. 2D-RMSD of the six first representative conformations of DA_8D peptide in protonated form ($DA_8D^{cMD}{}_{ASH}$ and $DA_8D^{cpHMD}{}_{pH1}$). RMSD is calculated using the C_α atoms of the peptides.

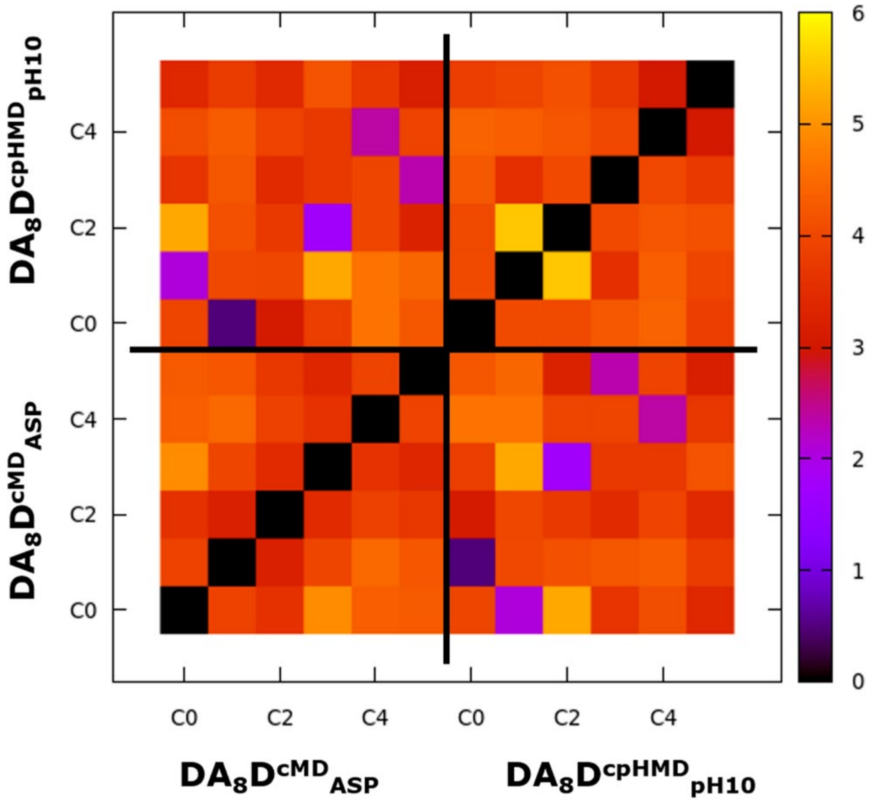

Figure S25. 2D-RMSD of the six first representative conformations of DA_8D peptide in the deprotonated form ($DA_8D^{cMD}{}_{ASP}$ and $DA_8D^{cpHMD}{}_{pH10}$). RMSD is calculated using the C_α atoms of the peptides.

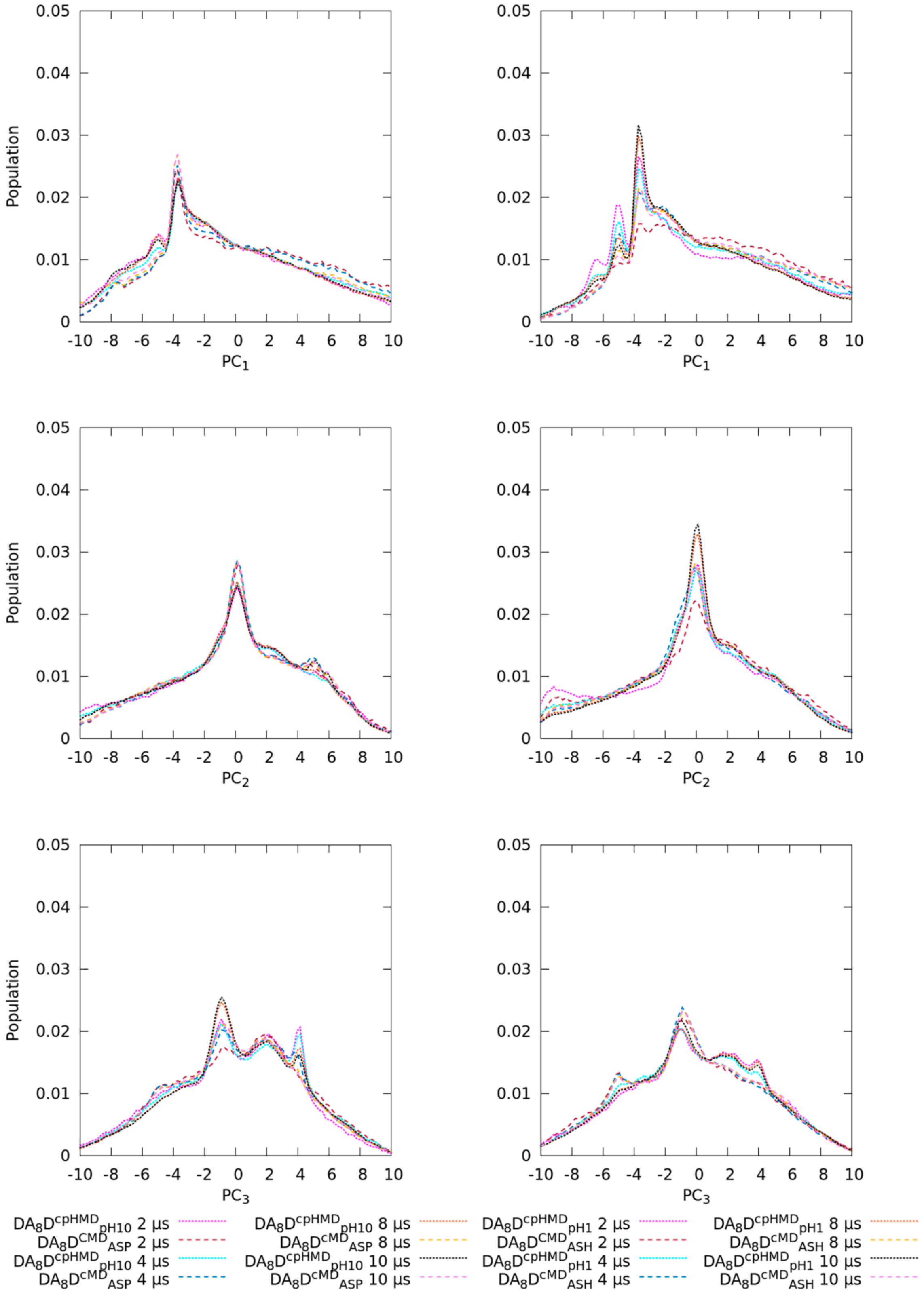

Figure S26. Distribution of the three first PC at several simulation times (2, 4, 8 and 10 µs) of the DA_8D peptide. Deprotonated and protonated form are in the left and right, respectively. Dotted and dashed lines are cpHMD and cMD simulation methods, respectively.

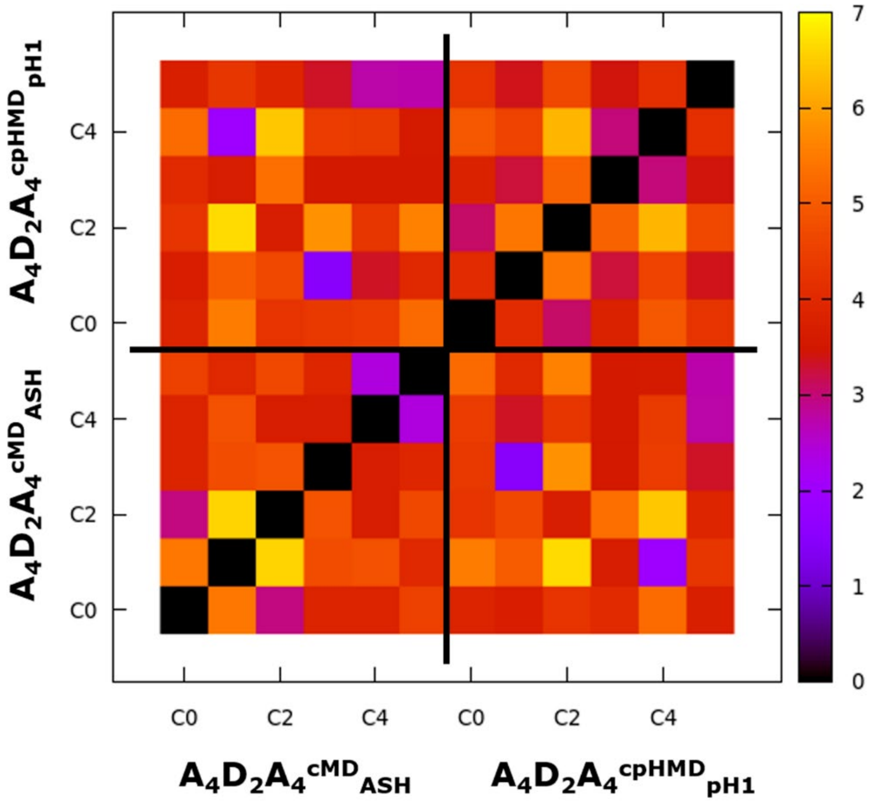

Figure S27. 2D-RMSD of the six first representative conformations of $A_4D_2A_4$ peptide in protonated form ($A_4D_2A_4{}^{cMD}{}_{ASH}$ and $A_4D_2A_4{}^{cpHMD}{}_{pH1}$). RMSD is calculated using the C_α atoms of the peptides.

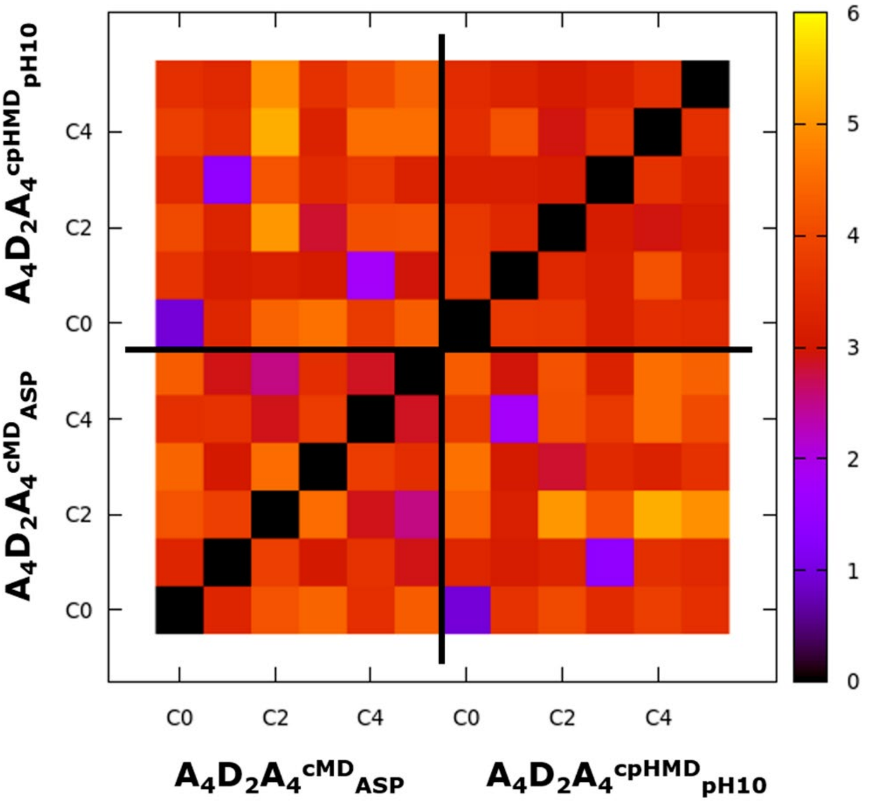

Figure S28. 2D-RMSD of the six first representative conformations of $A_4D_2A_4$ peptide in the deprotonated form ($A_4D_2A_4{}^{cMD}{}_{ASP}$ and $A_4D_2A_4{}^{cpHMD}{}_{pH10}$). RMSD is calculated using the C_α atoms of the peptides.

REFERENCES

1. Rubio-Martinez, J.; Tomas, M.S.; Perez, J.J. Effect of the solvent on the conformational behavior of the alanine dipeptide deduced from MD simulations. *J. Mol. Graph. Model.* **2017**, *78*, 118–128, doi:10.1016/j.jmgm.2017.10.005.
